# Sex-based disparities in postoperative outcomes after catheter ablation for atrial fibrillation: a systematic review and meta-analysis of propensity-matched studies

**DOI:** 10.1186/s12872-026-05942-2

**Published:** 2026-05-09

**Authors:** Ogechukwu Samuel Obi, Alysa Malik, Ilias Koziakas, Muhammad Hamza, Ahmed W. Hageen, Paweł Łajczak, Muhammad Khan, Uchenna Diane Nweze, Somtochukwu Susan Muogbo, Kamil Ahmad Kamil, Michel Pompeu Sá

**Affiliations:** 1 Lexington Medical Center, 145 Sunset Court, Suite 200, West Columbia, South Carolina USA; 2 Shifa College of Medicine, Islamabad, Pakistan; 3 Onassis Cardiac Surgery Center, Athens, Greece; 4 Saidu Medical College, Swat, Pakistan; 5 Faculty of Medicine, Tanta University, Tanta, Egypt; 6 Medical University of Silesia, Katowice, Poland; 7 Bacha Khan Medical College, Mardan, Pakistan; 8 Kaiser Permanente, Fontana Medical Center , Fontana, USA; 9 Millennium College of Nursing Science, Awka, Anambra State Nigeria; 10 Internal Medicine Department, Mirwais Regional Hospital, Kandahar, Afghanistan; 11 Department of Cardiovascular Surgery, Heart, Thoracic & Vascular Institute, Cleveland Clinic Florida, Weston, USA

**Keywords:** Atrial fibrillation, Catheter ablation, Sex differences, Female, Male, Arrhythmia recurrence

## Abstract

**Background and aim:**

Sex-related differences in outcomes after catheter ablation for atrial fibrillation (AF) remain incompletely understood, particularly regarding long-term arrhythmia recurrence and procedural safety. Current guidelines do not provide sex-specific recommendations. This systematic review and meta-analysis aimed to evaluate sex-based differences in efficacy, safety, and long-term outcomes following AF catheter ablation.

**Methods:**

We conducted a systematic review and meta-analysis in accordance with PRISMA guidelines and the Cochrane Handbook, with prospective registration in PROSPERO (CRD420251168422). PubMed, Embase, Web of Science, and the Cochrane Library were searched from inception to December 2025. Only studies adjusting for baseline differences using propensity score–based methods were included. Risk ratios (RRs) with 95% confidence intervals (CIs) were pooled using a restricted maximum likelihood random-effects model. Risk of bias was assessed using ROBINS-I, and certainty of evidence was evaluated using GRADE.

**Results:**

A total of 21 studies involving 103,619 patients undergoing AF ablation were included, of whom approximately 32% were female. There were no significant sex-based differences in AF recurrence at 1 year (RR 1.22, 95% CI [0.78, 1.91], I^2^ = 51.5%, τ²=0.0814, *p* = 0.3807), AF / atrial tachycardia (AT) recurrence at 1 year (RR 1.13, 95% CI [0.91, 1.39], I^2^ = 0%, τ²=0, *p* = 0.2659), AT / atrial flutter (AFL) / AT recurrence at 1 year (RR 1.03, 95% CI [0.68, 1.58], I^2^ = 79%, τ²=0.1067, *p* = 0.8744), early all-cause mortality (RR 1.07, 95% CI [0.50, 2.29], I^2^ = 0%, τ²=0, *p* = 0.8575), 1-year all-cause mortality (RR 0.49, 95% CI [0.14, 1.67], I^2^ = 56.4%, τ²=0.6668, *p* = 0.2523), follow-up stroke/TIA (RR 1.12, 95% CI [0.72, 1.76], I^2^ = 0%, τ²=0, *p* = 0.6107), follow-up repeat ablation (RR 1.03, 95% CI [0.86, 1.22], I^2^ = 0%, τ²=0, *p* = 0.7755). However, women had a higher risk of AF / AT recurrence at 2 years (RR 1.22, 95% CI [1.04, 1.42], I^2^ = 0.0%, τ²<0.0001, *p* = 0.0120), early complication rate (RR 1.37, 95% CI [1.10, 1.70], I^2^ = 59.6%, τ²=0.0368, *p* = 0.0051), early cardiac tamponade (RR 2.46, 95% CI [1.49, 4.07], I^2^ = 0.0%, τ²=0, *p* = 0.0004), and follow-up cardiac failure (RR 2.80, 95% CI [1.45, 5.38], I^2^ = 0%, τ²=0, *p* = 0.0020).

**Conclusion:**

Catheter ablation yields similar short-term rhythm control and mortality in women and men with AF. However, women have higher early procedural complications and worse long-term outcomes, including increased 2-year AF/AT recurrence and heart failure, highlighting the need for tailored strategies and closer follow-up in female patients.

**Supplementary Information:**

The online version contains supplementary material available at 10.1186/s12872-026-05942-2.

## Introduction

Atrial fibrillation (AF) is the most common sustained cardiac arrhythmia globally. It is associated with increased risks of stroke, heart failure, hospitalization, and mortality, as well as significant reductions in quality of life [1–3]. Catheter ablation has emerged as an effective rhythm-control strategy for symptomatic AF, particularly in patients who are refractory to antiarrhythmic drug therapy, and is increasingly considered as a first-line treatment in selected populations [4, 5].

Current guidelines from the American College of Cardiology (ACC), American Heart Association (AHA), Heart Rhythm Society (HRS), and European Society of Cardiology (ESC) recommend ablation based on clinical indication without sex-specific recommendations [6–8]. However, accumulating evidence indicates that female patients with AF may exhibit distinct patterns in presentation, disease progression, and procedural outcomes. Women are often older at the time of ablation, present later in the disease course, and may have more extensive atrial remodeling and fibrosis, which can predispose to higher rates of procedural complications and influence long-term arrhythmia recurrence [9, 10].

Early post-ablation AF recurrence appears comparable between sexes, likely driven by transient inflammation, lesion maturation, and autonomic changes. However, sex-related differences may emerge over longer follow-up, with women showing increased late recurrence of atrial arrhythmias and higher incidence of heart failure. Procedural complications also differ by sex: women experience higher rates of vascular complications, cardiac tamponade, pericardial effusion, and hematoma, although mortality and long-term rhythm-control strategies, including repeat ablation and antiarrhythmic drug therapy are generally similar between men and women [11–14]. Despite these findings, most studies are limited by underrepresentation of women, low event rates, and lack of prospective, sex-stratified analyses, leading to important uncertainties regarding the long-term impact of sex on AF ablation outcomes.

To address these limitations, this systematic review and meta-analysis comprehensively evaluates sex-based differences in outcomes following AF catheter ablation, including arrhythmia recurrence, procedural complications, mortality, and long-term management. Besides, we aim to determine whether female sex is associated with differences in procedural safety, short- and long-term rhythm outcomes, and post-ablation clinical events in women with AF.

## Methods

### Data sources

This systematic review and meta-analysis was conducted in accordance with the Cochrane Collaboration Handbook for Systematic Reviews of Interventions and complied with the Preferred Reporting Items for Systematic Reviews and Meta-Analyses (PRISMA) guidelines [15, 16]. The study protocol was prospectively registered in the International Prospective Register of Systematic Reviews (PROSPERO) under the registration number CRD420251168422. The ethical approval and institutional review board were waived because the investigators relied on the data from published articles. A completed PRISMA checklist is available in the Supplementary S1. As this is a systematic review and meta-analysis using secondary, de-identified data, it was not necessary to obtain patient consent or institutional review board approval.

### Eligibility criteria

In this analysis, we compared females and males undergoing catheter ablation for atrial fibrillation. We included studies that reported at least one outcome of interest. Studies utilizing propensity score matching, inverse probability of treatment weighting, or other propensity-based adjustment methods were considered eligible. (Supplementary S2). We excluded studies that involved in vitro analyses, case reports, animal studies, conference abstracts lacking complete data, review articles, studies with incomplete outcome reporting, studies with institutional overlap, and those involving patients undergoing concurrent cardiac procedures. Non-English studies were also omitted from our analysis.

### Search strategy and data extraction

We performed a literature search on PubMed, Embase, Scopus, Web of Science, and the Cochrane Library using the search strategy detailed in Supplementary S2. Studies were searched starting from inception to December 2025 with the following search terms: “atrial fibrillation”, “catheter ablation”, “radiofrequency ablation”, “cryoablation”, “sex characteristics”, “female”, “male”, “sex differences”, “sex-based differences”, and “sex-based”. The study selection process was independently performed by three reviewers (M. H., I. K., O. O.). A further manual search was conducted using the search strategy on Google Scholar, and the first 200 hits were screened. Additionally, it was applied to the references and citations of included studies to identify any studies that were missed during the primary search. Disagreements were resolved by consensus. After removing duplicates, the authors initially screened titles and abstracts and then assessed full-text articles for inclusion. We also manually screened the references of included studies for potential records. To minimize duplication, we assessed potential overlap by comparing study periods, centers, and author groups, and excluded overlapping datasets where identified. Data extraction was conducted using a standardized form by A. M. and S. M., and confirmed by O. O. We collected information on sample size, study design, patient baseline characteristics, and clinical outcomes. Corresponding authors were contacted by email in cases of missing data. Data were extracted from Kaplan-Meier curves [17].

### Endpoints and subgroup analysis

Primary outcomes were atrial arrhythmia recurrence, i.e. AF recurrence at 1-year, Atrial fibrillation / Atrial tachycardia (AF / AT) recurrence at 1-year and 2-year, and Atrial fibrillation / Atrial flutter / Atrial tachycardia (AF / AFL/ AT ) recurrence at 1-year. Early all-cause mortality within 30 days or hospital stay and at 1-year were assessed. Secondary outcomes were categorized into short-term and long-term outcomes. Short-term outcomes were defined as events that occurred during the hospital stay or within 30 days following the procedure. These outcomes included all-cause mortality, arteriovenous (AV) fistula, cardiac tamponade, complication rate, hematoma, pericardial effusion, phrenic nerve injury, pneumothorax or hemothorax, pulmonary complications, antiarrhythmic drug (AAD) use at discharge, Pulmonary vein isolation (PVI) success, and stroke/transient ischemic attack (TIA). Long-term outcomes were assessed after a minimum follow-up of 1 year and included AAD use at follow-up, cardiac failure follow-up, repeat ablation at follow-up, and stroke/TIA at follow-up. Due to variability in reported follow-up durations across included studies, outcomes were pooled using the longest reported follow-up from each study .

Prespecified subgroup analyses for ablation type, study design, and risk of bias were not performed due to the limited number of studies reporting each outcome.

### Risk of bias assessment and certainty of evidence

The assessment of risk of bias for the included studies was conducted independently by P. L. and O.O., using the Cochrane tool specifically designed for evaluating risk of bias in non-randomized studies (ROBINS-I), with disagreements resolved through consensus [18]. The studies were classified as having low, moderate, or serious risk of bias across various domains, which included confounding; participant selection; intervention classification; deviations from intended interventions; missing data; outcome measurement; and the selection of reported results. Any disagreements that arose were resolved through consensus.

The quality assessment results were displayed in plot form using the Robvis tool [19]. GRADE was used to assess the certainty of evidence [20]. Formal publication bias assessment was not performed due to fewer than 10 studies per outcome; however, this limitation is acknowledged.

### Statistical analysis

A pairwise meta-analysis was conducted using risk ratios (RR) with 95% confidence intervals (CIs). A two-tailed p-value below 0.05 was deemed statistically significant. The presence of statistical heterogeneity among studies was evaluated using Cochran’s Q test, Tau2, and I^2^ statistics. Heterogeneity was categorized as unimportant (I^2^ < 25%), moderate (25% < I^2^ < 50%), substantial (50% < I^2^ < 75%), and considerable (I^2^ > 75%). A Restricted Maximum Likelihood random-effects model was used to account for heterogeneity [21]. Publication bias was not assessed as no outcome was reported by at least 10 studies. RStudio, version 4.5.2 (The R Project for Statistical Computing, Vienna, Austria), was employed for the analysis. The meta package was used for statistical analysis [22–25]. The Luo and Wan methods were used for converting the median to the mean [26, 27].

### Sensitivity analyses

We conducted leave-one-out sensitivity analyses for all outcomes to evaluate how influential studies impacted the pooled analysis. Each study was removed sequentially, one by one, and the data were reanalyzed to verify the stability of the pooled effects.

## Results

### Study selection and characteristics

**Fig. 1:**
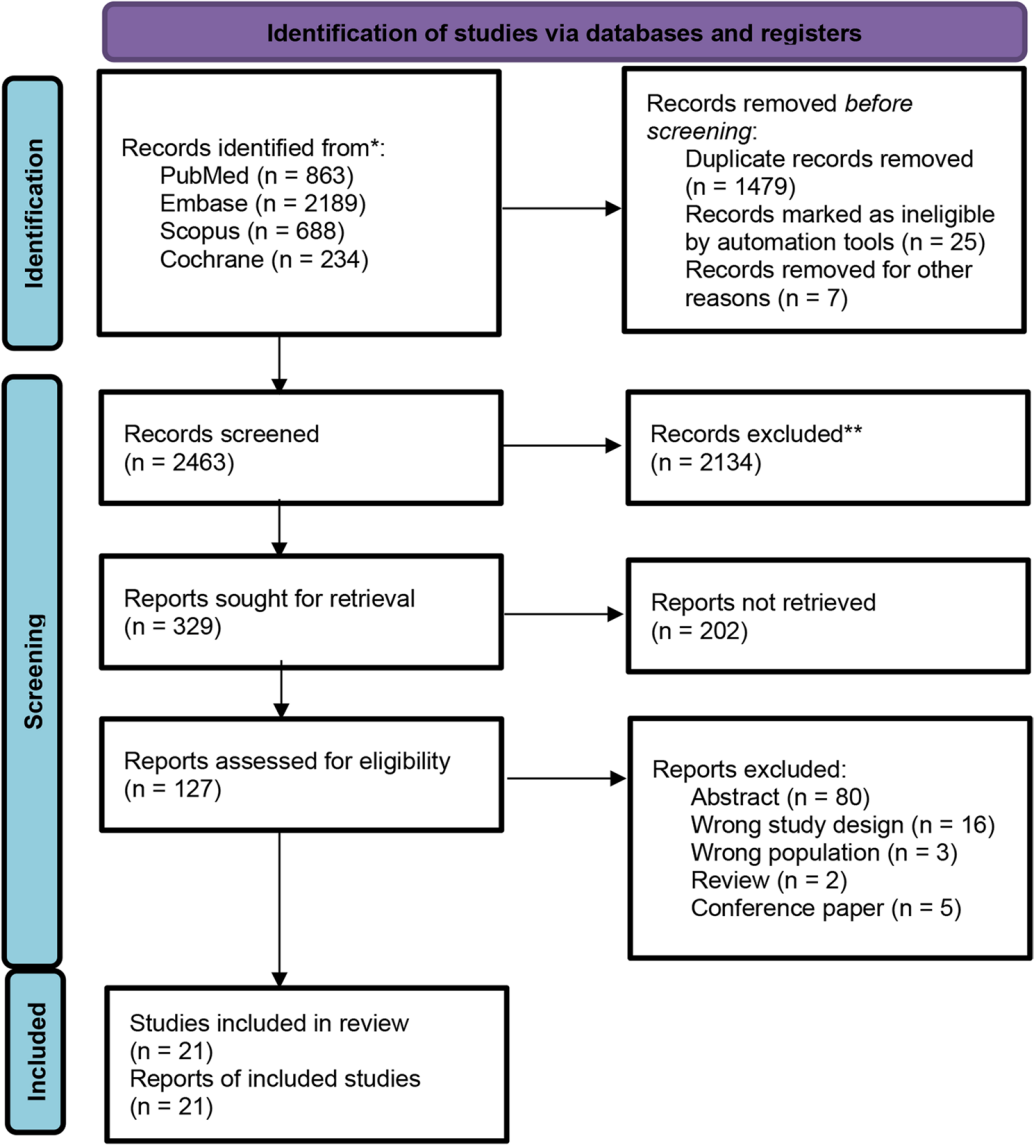
PRISMA Diagram

Our systematic search identified 3,974 potentially relevant articles (Fig. 1). Following the removal of duplicate records and exclusion based on title and abstract screening, 2463 studies remained for full-text review according to the inclusion and exclusion criteria. Of these, 21 studies met all the inclusion criteria. A total of 103,619 patients receiving catheter ablation for atrial fibrillation were analyzed, of whom 50,679 (48.9%) were female. The analysis included a population of 103,619 patients with an overall mean follow-up duration of 42.1 ± 13.6 months (Table 1). All included studies were 20 retrospective and 1 prospective studies published from 2015 to 2025.

**Table 1:** Baseline characteristics of included studies

Study ID	Retrospective / Prospective	Place Duration	Population	Denovo or Redo Ablation	No of Patients Female / Male	Age Years* Female / Male	BMI kg/m2* Female / Male	AF duration Months* Female / Male	CHA2DS2-VASc score* Female / Male	Paroxysmal AF *n* (%) Female / Male	Diabetes *n* (%) Female / Male	Hypertension *n* (%) Female / Male	AAD *n* (%) Female / Male	Follow - Up Months* Female / Male
Al-Sadawi et al. 2023 [28]	Retrospective	New York 2013–2021	CB or RF Catheter Ablation	Redo 93 (10.5)	444 / 444	70 (63–74)/ 69 (63–73)	30 (25–35) / 30 (27–33)	NA	NA	348 (78) / 359 (81)	70 (16) / 79 (18)	268 (60) / 275 (62)	Total 310 (70) / 300 (69)	NA
Chen et al. 2025 [29]	Retrospective	China 2020–2022	EIVOM	Redo 24 (9.1)	110 / 151	63.69 ± 8.46 / 62.68 ± 9.46	25.04 ± 3.77 / 25.18 ± 3.21	24 (6–60) / 12(3–60)	3 (2–4) / 2 (1–4)	47 (42.7) / 30 (19.9)	8 (7.3) / 21 (13.9)	72 (65.5) / 93 (61.6)	Amiodarone 39 (35.5) / 53 (35.1)	12 (12-16.5) / 12 (12-23.5)
Du et al. 2025 [30]	Retrospective	China 2013–2021	RF Catheter Ablation	Denovo Ablations	271 / 271	63.30 ± 9.26 / 62.93 ± 8.87	25.35 ± 3.75 / 25.19 ± 3.52	12.0 (4.0–45.0) / 12.0 (3.0–48.0)	NA	158 (58.30) / 168 (61.99)	35 (12.92) / 27 (9.96)	125 (46.13) / 121 (44.65)	NA	56.31 ± 22.32 / 56.34 ± 24.15
Duarte et al. 2025 [9]	Retrospective	Portugal 2019–2023	CB or RF Catheter Ablation	Denovo Ablations	113 / 113	63.0 (55.0–67.1) / 61.0 (54.0–67.3)	27.3 (24.2–30.5) / 27.4 (25.0-29.4)	30 (18–54) / 42 (18–63.6)	NA	91 (81) / 88 (78)	13 (12) / 13 (12)	58 (51) / 54 (48)	Total 74 (65) / 65 (58)	NA
Khelae et al. 2025 [31]	Prospective	37 countries 2016–2022	CB Catheter Ablation	Denovo Ablations	1136 / 1953	64 ± 10 / 59 ± 12	27 ± 5 / 28 ± 5	34.8 ± 49.2 / 39.6 ± 60	2.9 ± 1.4 / 1.4 ± 1.3	959 (84.4) / 1562 (80.0)	138 (12.1) / 267 (13.7)	685 (60.3) / 1005 (51.5)	NA	NA
Kim et al. 2021 [32]	Retrospective	South Korea 2006–2015	Catheter Ablation	NA	363 / 363	61.0 ± 8.1 / 62.5 ± 15.7	NA	22.8 (6.0-60.8) / 25.2 (7.7–58.9)	3.5 ± 1.7 / 3.5 ± 1.8	NA	54 (14.9) / 59 (16.3)	291 (80.2) / 304 (83.7)	Class Ic 238 (65.6) / 236 (65.0) Class III 109 (30.0) / 120 (33.1)	NA
Lin et al. 2025 [33]	Retrospective	China 2015–2020	CB or RF Catheter Ablation	NA	726 / 726	61.52 ± 9.68 / 60.75 ± 10.79	25.92 ± 4.01 / 26.12 ± 3.32	38.83 ± 40.85 / 35.29 ± 37.49	2.60 ± 1.48 / 1.57 ± 1.44	464 (63.9) / 469 (64.6)	123 (16.9) / 119 (16.4 )	366 (50.4) / 364 (50.1)	NA	50.11 ± 19.64 / 56.43 ± 19.75
Lv et al. 2025 [34]	Retrospective	China 2018–2021	CB or RF Catheter Ablation	NA	362 / 362	65.0 (56.0–72.0) / 64.0 (57.0–71.0)	25.0 (23.0–28.0) / 26.0 (23.0–28.0)	20.0 (12.0–27.0) / 20.0 (12.0–27.0)	3.0 (2.0–4.0) / 2.0 (1.0–2.0)	231 (63.81) / 220 (60.77)	60 (16.57) / 58 (16.02)	196 (54.14) / 191 (52.76)	NA	NA
Ma et al. 2023 [35]	Retrospective	China 2018–2020	CB or RF Catheter Ablation	Denovo Ablations	176 / 176	61.01 ± 10.54 / 61.10 ± 9.99	24.73 ± 3.38 / 24.78 ± 2.79	50.07 ± 54.36 / 46.72 ± 51.20	2.27 ± 1.14 / 1.15 ± 1.07	176 (100) / 176 (100)	24 (13.64) / 23 (13.07)	83 (47.16) / 71 (40.34)	Class Ic 94 (53.41) / 96 (54.55) Class II 34 (19.32) / 31 (17.61) Class III 58 (32.95) / 52 (29.55)	23.61 ± 13.21 / 24.45 ± 13.52
Mills et al. 2025 [36]	Retrospective	19 countries 2000–2024	Catheter Ablation	NA	34,622 / 34,622	66.9 ± 10.2 / 66.8 ± 9.9	NA	NA	NA	23,751 (68.6) / 23,578 (68.1)	6197 (17.9) / 5886 (17.0)	20,911 (60.4) / 20,531 (59.3)	Amiodarone 8794 (25.4) / 8482 (24.5)	NA
Ngo et al. 2021 [12]	Retrospective	Australia and New Zealand 2008–2017	Catheter Ablation	Redo 2367 (12.01)	9848 / 9848	64.7 ± 11.1 / 64.7 ± 10.8	NA	NA	NA	NA	909 (9.2) / 908 (9.2)	1329 (13.5) / 1275 (13.0)	NA	Overall 1 month
Pak et al. 2021 [37]	Retrospective	South Korea	RF Catheter Ablation	Redo 327 (100)	109 / 218	NA	NA	NA	NA	NA	NA	NA	NA	35.0 ± 28.6 / 36.4 ± 30.1
Park et al. 2023 [38]	Retrospective	South Korea 2009–2019	Catheter Ablation	Denovo Ablations	469 / 469	61.7 ± 9.9 / 61.8 ± 10.2	NA	NA	2.6 ± 1.5 / 1.6 ± 1.5	347 (74.0) / 340 (72.5)	72 (15.4) / 77 (16.4)	242 (51.6) / 211 (45.0)	NA	NA
Yu et al. 2018 [39]	Retrospective	South Korea 2009–2016	RF Catheter Ablation	Denovo Ablations	215 / 573	50.8 ± 8.4 / 50.1 ± 7.2	23.8 ± 3.4 / 25.3 ± 2.9	70.3 ± 172.0 / 82.3 ± 212.9	1.6 ± 0.8 / 0.6 ± 0.9	NA	11 (5.1) / 37 (6.5)	56 (26.0) / 169 (29.5)	NA	35.4 ± 22.5 / 32.9 ± 22.2
Ricciardi et al. 2019 [40]	Retrospective	Italy 2012	CB Catheter Ablation	NA	584 / 1541	62.4 ± 9.9 / 58.8 ± 10.6	27.1 ± 5.2 / 27.1 ± 3.6	52.8 ± 104.3 / 51.8 ± 65.0	NA	450 (77.1) / 1091 (70.8)	29 (4.8) / 86 (5.6)	298 (50.7) / 764 (49.6)	NA	Overall 16.8 ± 13.9 months
Ríos-Muñoz et al. 2022 [41]	Retrospective	Spain	RF Catheter Ablation	Denovo Ablations + Redo	21 / 16	65.2 ± 8.6 / 61.3 ± 9.5	NA	31.2 ± 33.6 / 31.2 ± 32.4	2.1 ± 0.0 / 1.6 ± 0.4	0 (0) / 0 (0)	6 (28.6) / 2 (12.5)	15 (71.4) / 9 (56.3)	NA	Overall 11.9 months
Roh et al. 2018 [42]	Retrospective	South Korea 1998–2014	RF Catheter Ablation	Denovo Ablations	367 / 367	59 ± 10 / 59 ± 10	25 ± 4 / 25 ± 3	57 ± 57 / 60 ± 58	NA	255 (69) / 255 (69)	29 (8) / 35 (10)	133 (36) / 124 (34)	Class I 132 (36) / 150 (41)Class III 102 (28) / 86 (23)	55 ± 38 / 55 ± 39
Shah et al. 2015 [43]	Retrospective	Illinois 2004–2012	Surgical ablation	NA	251 / 251	NA	NA	NA	NA	NA	NA	NA	NA	50.4 ± 28.8 / 46.8 ± 27.6
Turagam MK et al. 2023 [44]	Retrospective	24 European centers 2021–2022	PFA	Denovo Ablations	365 / 365	66.4 ± 10.1 / 66.5 ± 9.1	27.7 ± 5.4 / 27.9 ± 4.3	NA	2.8 ± 1.4 / 2.0 ± 1.4	256 (70.1) / 211 (57.8)	47 (12.9) / 49 (13.4)	219 (60.0) / 228 (62.5)	Class I 106 (29) / 95 (26.1) Class III 80 (21.9) / 78 (21.4)	NA
Veen et al. 2023 [45]	Retrospective	Netherlands 2012–2017	VATS PVI	NA	40 / 40	^62.0 ± 7.0 / 60.0 ± 7.71^	29.2 (25.7–32.5) / 29.2 (25.7–31.9)	NA	NA	22 (55) / 24 (60)	3 (7.50) / 4 (10.26)	15 (37.5) / 12 (30.8)	Total 38 (95) / 38 (95)	Overall 12 months
Zhu et al. 2022 [46]	Retrospective	China 2018–2021	RF Catheter Ablation	NA	87 / 71	70 ± 9 / 67 ± 11	29.40 ± 3.17 / 29.57 ± 2.31	NA	4 (3–5) / 3 (1–4)	48 (55.2) / 43 (60.6)	52 (60) / 27 (38)	56 (64) / 46 (65)	NA	14.8 (9.9–26.8)

*AAD* Antiarrhythmic drugs, *AF* Atrial fibrillation, *CB* Cryoballoon ablation, *EIVOM* Ethanol infusion of the vein of Marshall, *PFA* Pulsed field ablation, *RF* Radiofrequency ablation, *VATS* Surgical video assisted thoracoscopic, *PVI* Pulmonary vein isolation

*Data is presented as Mean ± Standard Deviation or Median (IQR)

These findings should be interpreted cautiously due to the limited number of contributing studies. Several outcomes were derived from a small number of studies, which may limit statistical power and precision.

### Primary outcomes

#### Atrial arrhythmias recurrence

##### Atrial fibrillation (AF) recurrence at 1-year

**Fig. 2:**
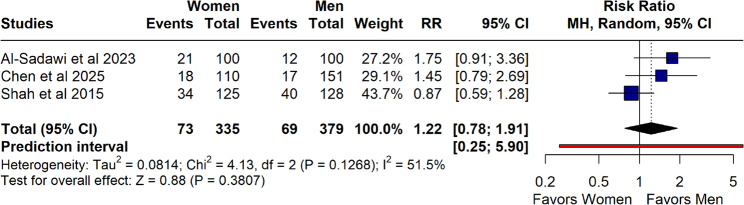
Atrial Fibrillation (AF) recurrence at 1-year

Three studies AF recurrence at 1-year and demonstrated no significant sex-based differences (RR 1.22, 95% CI [0.78, 1.91], I^2^ = 51.5%, τ²=0.0814, *p* = 0.3807, Fig. 2), which was supported by a wide prediction interval (0.25–5.90). Sensitivity analysis revealed that the substantial heterogeneity was largely driven by the study by Shah et al., 2015, as omission of this study reduced the I² statistic to 0% (Supplementary S5a).

##### Atrial Fibrillation / Atrial Tachycardia (AF / AT) recurrence at 1-year

**Fig. 3:**
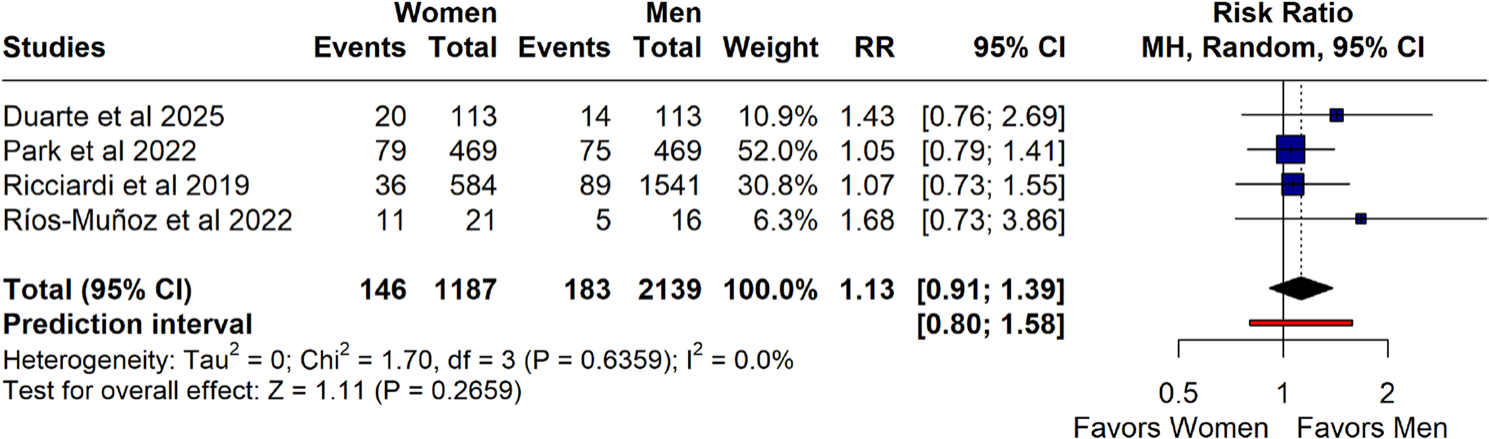
Atrial Fibrillation / Atrial Tachycardia (AF / AT) recurrence at 1-year

Four studies reported AF / AT recurrence at 1-year and found no significant sex-based differences (RR 1.13, 95% CI [0.91, 1.39], I^2^ = 0%, τ²=0, *p* = 0.2659, Fig. 3), which was supported by the prediction interval of (0.80–1.58).

##### Atrial Fibrillation / Atrial Tachycardia (AF / AT) recurrence at 2-years

**Fig. 4:**
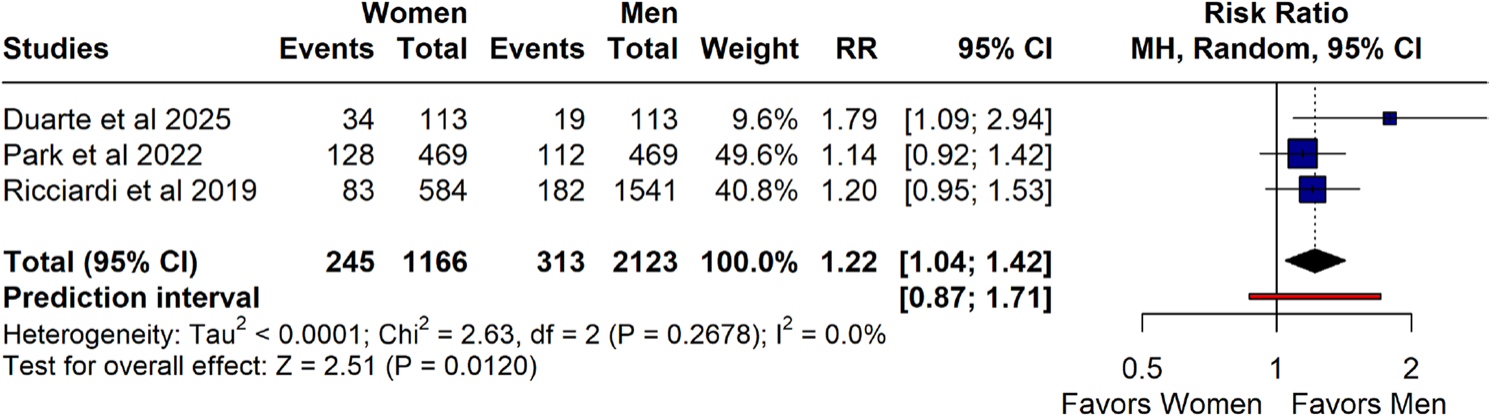
Atrial fibrillation / Atrial tachycardia (AF / AT) recurrence at 2-years

Three studies reported AF / AT recurrence at 2 years and demonstrated that women have a significantly higher likelihood of AF / AT recurrence compared to men (RR 1.22, 95% CI [1.04, 1.42], I^2^ = 0%, τ²<0.0001, *p* = 0.0120, Fig. 4). However, the prediction interval was wide and crossed unity (0.87–1.71), suggesting that the effect may vary in future populations.

##### Atrial Fibrillation / Atrial Flutter / Atrial Tachycardia (AF / AFL/ AT ) recurrence at 1-year

**Fig. 5:**
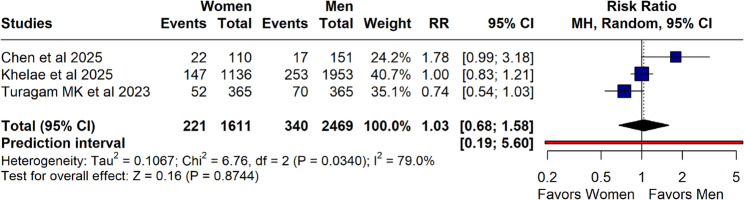
Atrial fibrillation / Atrial flutter / Atrial tachycardia (AF / AFL/ AT ) recurrence at 1-year

Three studies reported AF/AFL/AT recurrence at 1-year and demonstrated no significant sex-based differences (RR 1.03, 95% CI [0.68, 1.58], I^2^ = 79%, τ²=0.1067, *p* = 0.8744, Fig. 5). The prediction interval was wide and crossed unity (0.19–5.60), indicating that the true effect may vary considerably across settings. Leave-one-out analysis demonstrated consistent results, with no individual study exerting a disproportionate influence on the pooled estimate. The association remained non-significant across all exclusion analyses, supporting the robustness of the primary analysis despite residual heterogeneity (Supplementary S5b).

#### All-cause mortality

##### Early all-cause mortality

**Fig. 6:**
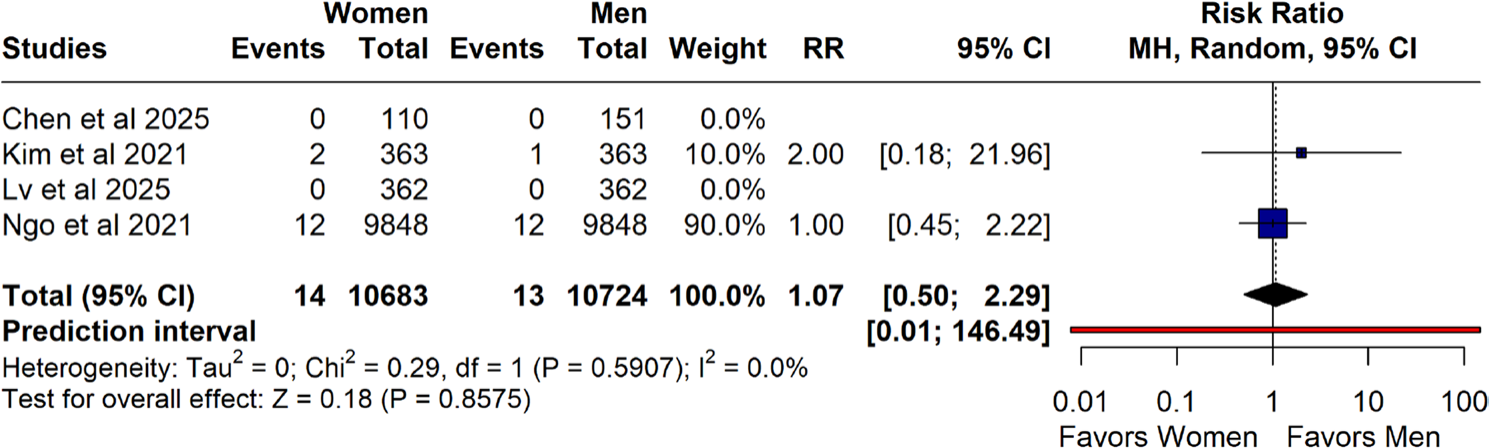
Early all-cause mortality

Four studies reported early all-cause mortality, and there was no significant difference between women and men (RR 1.07, 95% CI [0.50, 2.29], I^2^ = 0%, τ²=0, *p* = 0.8575, Fig. 6). This was corroborated by a wide prediction interval (0.01–146.49), reflecting marked imprecision in the pooled estimate.

##### 1-year all-cause mortality

**Fig. 7:**
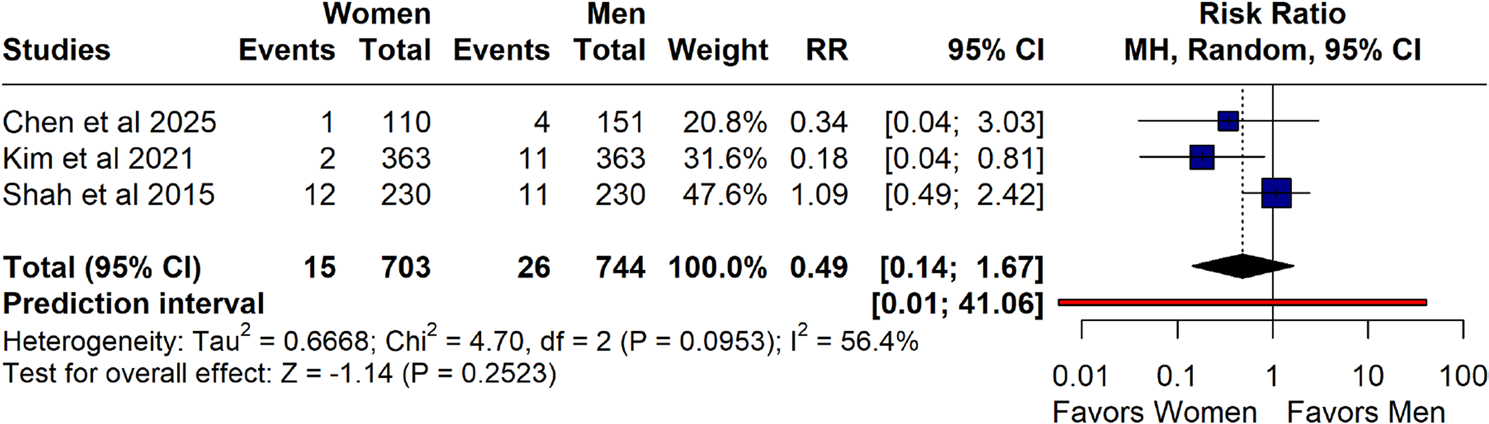
1-year all-cause mortality

Three studies reported 1-year all-cause mortality and demonstrated no significant difference between women and men (RR = 0.49, 95% CI [0.14, 1.67], I²=56.4%, τ²=0.6668, *p* = 0.2523, Fig. 7), which was supported by a wide prediction interval (0.01–41.06). Sensitivity analysis indicated that the observed heterogeneity was primarily driven by the studies by Kim et al., 2021 and Shah et al., 2015, as exclusion of these studies reduced the I² statistic to 0%, suggesting they were the main contributors to between-study variability (Supplementary S5c).

### Secondary outcomes

#### Short-term outcomes

##### Early AV fistula

Three studies reported early AV fistula and demonstrated no statistically significant differences between women and men (RR 0.60, 95% CI [0.12, 2.89], I^2^ = 0.0%, τ²=0, *p* = 0.5251, Supplementary S3a). The absence of a statistically significant difference was reinforced by a wide prediction interval (0.02–18.95), suggesting considerable variability in the estimated effect across potential future populations.

##### Early cardiac tamponade

Five studies reported early cardiac tamponade and showed that early cardiac tamponade was significantly higher in women as compared to men (RR 2.46, 95% CI [1.49, 4.07], I^2^ = 0.0%, τ²=0, *p* = 0.0004, Supplementary S3b). This finding was supported by the prediction interval (1.21–5.01), indicating consistency of the effect across future studies.

##### Early complication rate

Six studies reported early complication rate and showed that early complication rate was significantly increased in women compared to men (RR 1.37, 95% CI [1.10, 1.70], I^2^ = 59.6%, τ²=0.0368, *p* = 0.0051, Supplementary S3c). This was confirmed by a prediction interval of [0.77, 2.42]. Sensitivity analysis revealed that the substantial heterogeneity was largely driven by the study by Ngo et al., 2021, as omission of this study reduced the I² statistic to 0% (Supplementary S5d).

##### Early hematoma

Four studies reported the outcome of early hematoma and demonstrated significantly increased incidence of early hematoma in women compared to men (RR 1.25, 95% CI [1.06, 1.46], I^2^ = 0%, τ²=0, *p* = 0.0063, Supplementary S3d). This was confirmed by a prediction interval of [0.96, 1.61].

##### Early pericardial effusion

Six studies reported early pericardial effusion and demonstrated significantly increased incidence of early pericardial effusion in women compared to men (RR 1.35, 95% CI [1.08, 1.67], I^2^ = 24.1%, τ²=0.0166, *p* = 0.0069, Supplementary S3e). This was confirmed by the prediction intervals of [0.87, 2.08].

##### Early phrenic nerve injury

Six studies reported the outcome of early phrenic nerve injury and showed no significant differences between women and men (RR 1.60, 95% CI [0.91, 2.81], I^2^ = 8.3%, τ²=0.0346, *p* = 0.1060, Supplementary S3f). This was consistent with a prediction interval of [0.53, 4.77].

##### Early pneumothorax or hemothorax

Three studies reported early pneumothorax or hemothorax and showed no significant differences between women and men (RR 1.07, 95% CI [0.52, 2.22], I^2^ = NA, τ²=NA, *p* = 0.8526, Supplementary S3g).

##### Early pneumonia

Three studies reported pneumonia and showed no statistically significant sex-based differences (RR 0.60, 95% CI [0.22, 1.69], I^2^ = 48.7%, τ²=0.4404, *p* = 0.3381, Supplementary S3h). Leave-one-out sensitivity analysis showed consistent findings, with exclusion of individual studies not changing the overall non-significant effect. Although heterogeneity increased when Ma et al. 2023 was omitted (I^2^ = 72.9%), the pooled estimate remained non-significant (Overall RR 0.60, 95% CI [0.22, 1.69], I^2^ = 48.7%, *p* = 0.3381) (Supplementary S5e).

##### Early stroke/TIA

Five studies reported early stroke/TIA, and it showed no significant differences between women and men (RR 0.82, 95% CI [0.50, 1.34], I^2^ = 0%, τ²=0, *p* = 0.4278, Supplementary S3i). This was consistent with a prediction interval of [0.37, 1.82].

##### Early vascular complications

Three studies reported pseudo-aneurysm or aneurysm and demonstrated no significant sex-based differences (RR 1.48, 95% CI [0.38, 5.80], I^2^= 0%, τ²=0, *p* = 0.5765, Supplementary S3j). Across four studies, there was no statistically significant difference in the risk of vascular complications between women and men (RR = 1.38, 95% CI [0.99, 1.93], I² = 0%, τ²=0, *p* = 0.054; Supplementary S3j).

##### Early PVI success

Three studies reported early PVI success, and it showed that it’s not statistically significant between women and men (RR 1.00, 95% CI [0.99, 1.01], I^2^ = 0%, τ²=0, *p* = 0.6247, Supplementary S3k). The narrow prediction interval (0.97–1.02) suggests minimal between-study variability and a consistent absence of a sex-based difference.

##### AAD use at discharge

Three studies reported AAD use at discharge and demonstrated no statistically significant sex-based differences (RR 1.01, 95% CI [0.90, 1.13], I^2^ = 0%, τ²=0, *p* = 0.9248, Supplementary S3l). This is consistent with a prediction interval of [0.78–1.29].

#### Long-term outcomes

##### Follow-up stroke/TIA

Five studies reported follow-up stroke/TIA with no significant sex-based differences (RR = 1.12, 95% CI [0.72, 1.76], I² = 0%, τ²=0, *p* = 0.6107, Supplementary S4a), and the prediction interval (0.60–2.12) suggested consistency of effect across existing and potential future studies.

##### Follow-up repeat ablation

Four studies reported follow-up repeat ablation with no significant sex-based differences (RR 1.03, 95% CI [0.86, 1.22], I^2^ = 0%, τ²=0, *p* = 0.7755, Supplementary S4b), and the prediction interval (0.77, 1.36) suggests consistent effects across future studies.

##### Follow-up cardiac failure

Four studies reported follow-up cardiac failure and demonstrated statistically increased incidence of follow-up cardiac failure in women compared to men (RR 2.80, 95% CI [1.45, 5.38], I^2^ = 0%, τ²=0, *p* = 0.0020, Supplementary S4c), with a prediction interval of 0.97–8.09, indicating uncertainty in the expected effect size in individual future studies despite low between-study heterogeneity.

##### Follow-up AAD

Three studies reported follow-up AAD use and demonstrated no significant sex-based differences (RR 0.97, 95% CI [0.80, 1.18], I² = 0%, τ²<0.0001, *p* = 0.7734, Supplementary S4d); the prediction interval (0.64–1.49) suggests consistent effects across future settings.

### GRADE

One-year atrial fibrillation recurrence was assigned very low certainty, mainly due to serious risk of bias and substantial inconsistency among the included non-randomized studies. Similarly, recurrence of atrial fibrillation, atrial tachycardia, or atrial flutter at one year was graded as very low certainty, reflecting methodological limitations and heterogeneity across studies.

One-year atrial fibrillation or atrial tachycardia recurrence and two-year recurrence outcomes were judged as having low certainty, primarily because of serious risk of bias inherent to observational study designs despite relatively consistent effect estimates.

Thirty-day all-cause mortality was also rated as low certainty, largely due to study design limitations, although effect estimates suggested little to no difference between females and males undergoing catheter ablation. Overall, most outcomes were downgraded because of serious risk of bias, with additional downgrading for inconsistency where heterogeneity was present. A summary of the GRADE certainty assessment is presented in Supplementary S6.

### Risk of bias

A total of 11 studies were graded with serious concerns, and 10 with moderate concerns. Most risk of bias was found in confounding, measurement of outcomes, and selection of participants (Supplementary S7).

## Discussion

This systematic review and meta-analysis evaluated sex-based differences in outcomes following catheter ablation for AF. A total of 21 studies, including 103,619 patients undergoing AF ablation, were analyzed. The primary aim of this study was to assess whether female sex is associated with differences in arrhythmia recurrence, mortality, procedural success, and short- and long-term complications compared with male patients.

The main findings of this study are as follows:

- No significant sex differences in short-term arrhythmia recurrence
- Higher 2-year AF/AT recurrence in women
- Higher rates of early procedural complications in women
- No differences in mortality outcomes
- Increased long-term heart failure risk in women

Current ACC/AHA/HRS and ESC guidelines endorse catheter ablation as an effective rhythm-control strategy for symptomatic atrial fibrillation, either after antiarrhythmic drug failure or as first-line therapy in selected patients, but do not offer sex-specific ablation recommendations [6, 47]. These findings may inform future updates to current ACC/AHA/HRS and ESC guidelines by highlighting the importance of sex-specific considerations in risk stratification and procedural planning. Although these guidelines and the EHRA/HRS/APHRS/LAHRS expert consensus acknowledge that female sex affects AF presentation, referral timing, complication risk, symptom burden, and quality of life, ablation decisions are based on clinical indication rather than sex, due to limited randomized, sex-stratified evidence [48–51]. This evidence suggests that later referral, higher procedural complications, and possibly higher long-term recurrence in women, highlighting an important evidence gap and the need for individualized decision-making and future sex-focused research. Women are frequently referred later for catheter ablation, often at an older age and with more advanced atrial remodeling and comorbidity burden [52]. This referral bias may contribute to the higher complication rates observed. Importantly, when baseline differences are minimized using propensity-matched analyses, outcomes tend to be more comparable between sexes, as demonstrated in other interventional cardiology settings. These findings support the need for equitable and timely referral for ablation in women.

Our meta-analysis aligns with the aim of current guidelines and evidence to guide the safe and effective use of catheter ablation to improve symptoms and quality of life in patients with AF. Our findings can be summarized as follows. First, there were no significant sex-based differences in AF recurrence at one year or in composite atrial arrhythmia recurrence (AF/AT or AF/AFL/AT) at one year. However, at two years, female patients had a significantly higher risk of AF/AT recurrence compared with males. Second, early and one-year all-cause mortality did not differ significantly between sexes. Third, female patients experienced higher rates of several early procedural complications, including cardiac tamponade, overall complication rate, hematoma, and pericardial effusion. Finally, during follow-up, women had a significantly higher incidence of heart failure, while no sex differences were observed in stroke/TIA, repeat ablation, antiarrhythmic drug use, or long-term procedural success.

The absence of significant sex differences in one-year AF recurrence aligns with several prior studies suggesting comparable short-term rhythm control between women and men after catheter ablation [35, 44, 53]. Early post-ablation recurrence is often driven by transient inflammation, lesion maturation, and autonomic changes, which may not differ substantially by sex [54–57]. The consistency of findings across multiple recurrence definitions at one year supports the strength of this observation. Our study documented higher risk of AF/AT recurrence in women at two years, which suggests that sex-related differences may emerge over longer follow-up. This finding is biologically plausible and has been reported in previous cohorts [33, 58–60]. Female patients undergoing AF ablation are often older and may have a higher burden of atrial fibrosis and more advanced disease at presentation, which could contribute to late recurrence. In addition, hormonal influences and differences in atrial substrate remodeling may play a role; however, these mechanisms remain speculative and should be interpreted as hypothesis-generating rather than causal [9, 50, 61, 62]. In addition, hormonal influences and differences in atrial substrate remodeling may contribute to progressive arrhythmogenicity over time [29, 63, 64]. Although the pooled estimate was statistically significant, the wide prediction interval crossing unity indicates that this effect may not be uniform across all populations, highlighting the need for individualized interpretation.

It has been relatively well-documented that women undergoing catheter ablation for AF experience higher rates of vascular/groin complications, bleeding complications, and pericardial effusion/tamponade [14, 65, 66]. However, most studies have been underpowered to detect differences in less commonly occurring major complications. The data in this analysis demonstrate no sex differences were observed in long-term stroke/TIA. Interestingly, prior meta‐analysis of catheter ablation for AF outcomes stratified by sex which showed significantly higher rates of stroke/TIA and mortality following catheter ablation for AF in women [58]. However, serious complications like stroke/TIA are rare, so studies often don’t show differences, even though women may be at higher long-term risk. Furthermore, mortality outcomes were similar between sexes, both early and at one year. This finding is consistent with prior studies showing that catheter ablation is a relatively safe procedure with low short-term mortality regardless of sex [10, 67]. However, the wide prediction intervals and low certainty of evidence reflect limited event rates and imprecision, suggesting that small differences cannot be definitively excluded.

A major finding of this study is the consistently higher rate of early procedural complications among female patients. The significantly increased risk of cardiac tamponade in women has been repeatedly reported in the literature and is likely multifactorial [11, 12, 68]. Smaller cardiac chamber size, thinner atrial walls, and differences in vascular anatomy have been proposed as potential contributors to increased susceptibility to perforation and pericardial injury; however, these explanations are based on observational and mechanistic data and should be considered hypothesis-generating [69, 70]. Similarly, the higher incidence of hematoma and pericardial effusion may be related to smaller vessel caliber, differences in anticoagulation response, and challenges with vascular access. Despite these higher complication rates, early PVI success was identical between sexes, indicating that procedural efficacy is not compromised in women. This reinforces the concept that women derive similar electrophysiological benefits from ablation when the procedure is successfully performed. Differences in procedural complexity, including catheter size, vascular access challenges, and operator-related factors, may also contribute to the higher complication rates observed in women.

In the long term, the higher incidence of heart failure among women is an important observation. Women with AF may be more likely to develop heart failure with preserved ejection fraction, and recurrent atrial arrhythmias could potentially exacerbate diastolic dysfunction and symptoms; however, these relationships cannot be definitively established from the current analysis and should be interpreted cautiously [71–73]. This finding may also reflect a higher baseline comorbidity burden or differences in post-ablation management and follow-up. Importantly, no sex differences were observed in repeat ablation and antiarrhythmic drug use, suggesting comparable overall disease control strategies after the initial procedure.

### Clinical implications and future directions

These findings have several important clinical implications. First, catheter ablation provides similar short-term rhythm outcomes in women and men, supporting equitable referral for ablation irrespective of sex. Second, the increased risk of procedural complications in women underscores the need for heightened procedural awareness, meticulous vascular access, and careful periprocedural management, particularly regarding anticoagulation and imaging guidance. Third, the higher risk of late arrhythmia recurrence and heart failure in women highlights the importance of long-term follow-up and comprehensive risk factor modification. Earlier referral for ablation, before advanced atrial remodeling occurs, may improve long-term outcomes in female patients. Clinicians should also maintain a lower threshold for post-ablation surveillance in women, particularly those with symptoms suggestive of heart failure.

Future research should focus on prospective, adequately powered studies specifically designed to explore sex-related differences in AF ablation outcomes. Mechanistic studies evaluating atrial fibrosis, autonomic regulation, and hormonal influences may provide further insight into the observed long-term differences. In addition, sex-specific risk stratification tools and tailored ablation strategies could help reduce complication rates and improve durability of rhythm control in women. Randomized trials and high-quality registries with standardized definitions and longer follow-up are needed to improve the certainty of evidence, as most outcomes in this analysis were graded as low or very low certainty.

It is important to note that the mechanistic explanations discussed above are derived from observational and translational data, and should be interpreted as hypothesis-generating rather than definitive causal pathways.

### Strengths and limitations

This study has several strengths. It provides a comprehensive synthesis of contemporary data, includes a large pooled population, and evaluates a wide range of clinically relevant outcomes with both short- and long-term follow-up. The use of sensitivity analyses and prediction intervals enhances the interpretability and robustness of the findings.

However, several limitations must be acknowledged. All included studies were observational, leading to a high risk and moderate risk of confounding and selection bias. Differences in patient characteristics, ablation techniques, operator experience, and follow-up protocols may have influenced outcomes. Heterogeneity was present for several endpoints, and some analyses were limited by small numbers of events and wide confidence intervals. Finally, sex-specific baseline characteristics could not be fully adjusted across studies.

Additionally, variability in AF subtype (paroxysmal versus persistent) and differences in ablation modalities (radiofrequency, cryoballoon, pulsed-field ablation) were not consistently reported across studies and may have influenced outcomes.

## Conclusion

In this systematic review and meta-analysis, women and men undergoing catheter ablation for AF demonstrated similar short-term rhythm outcomes and mortality. However, female patients experienced higher rates of early procedural complications, higher long-term arrhythmia recurrence, and increased risk of heart failure during follow-up. These findings highlight the need for differentiated management strategies and closer long-term follow-up in women. Future high-quality studies are needed to refine sex-specific strategies and improve outcomes for all patients undergoing AF ablation.

## Supplementary Information

Supplementary Material 1.

## Data Availability

All data analyzed during this study are included in this published article and its supplementary information files. Additional data are available from the corresponding author upon reasonable request.

## Abbreviations

AF: Atrial fibrillation
ACC: American College of Cardiology
AHA: American Heart Association
HRS: Heart Rhythm Society
ESC: European Society of Cardiology
PRISMA: Preferred Reporting Items for Systematic Reviews and Meta-Analyses
PROSPERO: Prospectively registered in the International Prospective Register of Systematic Reviews
AF / AT: Atrial fibrillation / Atrial tachycardia
AF / AFL/ AT: Atrial fibrillation / Atrial flutter / Atrial tachycardia
AAD: Antiarrhythmic drug
PVI: Pulmonary vein isolation
ROBINS-I: Risk of bias in non-randomized studies
