# Supplementary material for: Sex-based disparities in postoperative outcomes after catheter ablation for atrial fibrillation: a systematic review and meta-analysis of propensity-matched studies
Source: BMC Cardiovasc Disord. 2026 May 9;26:560. doi: 10.1186/s12872-026-05942-2 (PMC13330058; doi:10.1186/s12872-026-05942-2)
Supplement: Supplementary file 1 — Supplementary Material 1. [file 12872_2026_5942_MOESM1_ESM.docx]

**Supplemental Material**

***Gender-Based Disparities in Postoperative Outcomes After Catheter Ablation for Atrial Fibrillation: A Systematic Review and Meta-analysis of Propensity-Matched Studies***

**Supplemental Files Index**

**Supplementary S1 -** Preferred Reporting Items for Systematic Reviews and Meta-Analysis (PRISMA) checklist.

**Supplementary S2-** Research Question, PICOTT and Search Strategy

**Supplementary S3**- Forest plots for early secondary outcomes

**Supplementary S4**- Forest plots for long term secondary outcomes

**Supplementary S5**- Leave one out sensitivity analysis

**Supplementary S6-** GRADE certainty assessment

**Supplementary S7-** Quality assessment using ROBINS-I

**Supplementary S8-** Propensity Score Matching (PSM) Protocol Summary Table

**Supplementary S1 -** Preferred Reporting Items for Systematic Reviews and Meta-Analysis (PRISMA) checklist

**PRISMA checklist**

| **Section and Topic** | **Item #** | **Checklist item** | **Page where item is reported** |
| --- | --- | --- | --- |
| **TITLE** | | |  |
| Title | 1 | Identify the report as a systematic review. | 1 |
| **ABSTRACT** | | |  |
| Abstract | 2 | See the PRISMA 2020 for Abstracts checklist. | 2 |
| **INTRODUCTION** | | |  |
| Rationale | 3 | Describe the rationale for the review in the context of existing knowledge. | 3,4 |
| Objectives | 4 | Provide an explicit statement of the objective(s) or question(s) the review addresses. | 3,4 |
| **METHODS** | | |  |
| Eligibility criteria | 5 | Specify the inclusion and exclusion criteria for the review and how studies were grouped for the syntheses. | 5 |
| Information sources | 6 | Specify all databases, registers, websites, organisations, reference lists and other sources searched or consulted to identify studies. Specify the date when each source was last searched or consulted. | 5 |
| Search strategy | 7 | Present the full search strategies for all databases, registers and websites, including any filters and limits used. | S2 |
| Selection process | 8 | Specify the methods used to decide whether a study met the inclusion criteria of the review, including how many reviewers screened each record and each report retrieved, whether they worked independently, and if applicable, details of automation tools used in the process. | 5 |
| Data collection process | 9 | Specify the methods used to collect data from reports, including how many reviewers collected data from each report, whether they worked independently, any processes for obtaining or confirming data from study investigators, and if applicable, details of automation tools used in the process. | 5 |
| Data items | 10a | List and define all outcomes for which data were sought. Specify whether all results that were compatible with each outcome domain in each study were sought (e.g. for all measures, time points, analyses), and if not, the methods used to decide which results to collect. | 6 |
|  | 10b | List and define all other variables for which data were sought (e.g. participant and intervention characteristics, funding sources). Describe any assumptions made about any missing or unclear information. | 6 |
| Study risk of bias assessment | 11 | Specify the methods used to assess risk of bias in the included studies, including details of the tool(s) used, how many reviewers assessed each study and whether they worked independently, and if applicable, details of automation tools used in the process. | 6,7 |
| Effect measures | 12 | Specify for each outcome the effect measure(s) (e.g. risk ratio, mean difference) used in the synthesis or presentation of results. | 7 |
| Synthesis methods | 13a | Describe the processes used to decide which studies were eligible for each synthesis (e.g. tabulating the study intervention characteristics and comparing against the planned groups for each synthesis (item #5)). | 7 |
|  | 13b | Describe any methods required to prepare the data for presentation or synthesis, such as handling of missing summary statistics, or data conversions. | 7 |
|  | 13c | Describe any methods used to tabulate or visually display results of individual studies and syntheses. | 7 |
|  | 13d | Describe any methods used to synthesize results and provide a rationale for the choice(s). If meta-analysis was performed, describe the model(s), method(s) to identify the presence and extent of statistical heterogeneity, and software package(s) used. | 7 |
|  | 13e | Describe any methods used to explore possible causes of heterogeneity among study results (e.g. subgroup analysis, meta-regression). | 7 |
|  | 13f | Describe any sensitivity analyses conducted to assess robustness of the synthesized results. | 7 |
| Reporting bias assessment | 14 | Describe any methods used to assess risk of bias due to missing results in a synthesis (arising from reporting biases). | 6 |
| Certainty assessment | 15 | Describe any methods used to assess certainty (or confidence) in the body of evidence for an outcome. | 7 |
| **RESULTS** | | |  |
| Study selection | 16a | Describe the results of the search and selection process, from the number of records identified in the search to the number of studies included in the review, ideally using a flow diagram. | 8 |
|  | 16b | Cite studies that might appear to meet the inclusion criteria, but which were excluded, and explain why they were excluded. | 8 |
| Study characteristics | 17 | Cite each included study and present its characteristics. | 9 |
| Risk of bias in studies | 18 | Present assessments of risk of bias for each included study. | S7 |
| Results of individual studies | 19 | For all outcomes, present, for each study: (a) summary statistics for each group (where appropriate) and (b) an effect estimate and its precision (e.g. confidence/credible interval), ideally using structured tables or plots. | NA |
| Results of syntheses | 20a | For each synthesis, briefly summarise the characteristics and risk of bias among contributing studies. | 23 |
|  | 20b | Present results of all statistical syntheses conducted. If meta-analysis was done, present for each the summary estimate and its precision (e.g. confidence/credible interval) and measures of statistical heterogeneity. If comparing groups, describe the direction of the effect. | 12-22 |
|  | 20c | Present results of all investigations of possible causes of heterogeneity among study results. | 12-22 |
|  | 20d | Present results of all sensitivity analyses conducted to assess the robustness of the synthesized results. | 12-22 |
| Reporting biases | 21 | Present assessments of risk of bias due to missing results (arising from reporting biases) for each synthesis assessed. | 23, S7 |
| Certainty of evidence | 22 | Present assessments of certainty (or confidence) in the body of evidence for each outcome assessed. | 23, S6 |
| **DISCUSSION** | | |  |
| Discussion | 23a | Provide a general interpretation of the results in the context of other evidence. | 23-26 |
|  | 23b | Discuss any limitations of the evidence included in the review. | 27-28 |
|  | 23c | Discuss any limitations of the review processes used. | 27-28 |
|  | 23d | Discuss implications of the results for practice, policy, and future research. | 27 |
| **OTHER INFORMATION** | | |  |
| Registration and protocol | 24a | Provide registration information for the review, including register name and registration number, or state that the review was not registered. | 4 |
|  | 24b | Indicate where the review protocol can be accessed, or state that a protocol was not prepared. | 4 |
|  | 24c | Describe and explain any amendments to information provided at registration or in the protocol. | NA |
| Support | 25 | Describe sources of financial or non-financial support for the review, and the role of the funders or sponsors in the review. | 29 |
| Competing interests | 26 | Declare any competing interests of review authors. | 29 |
| Availability of data, code and other materials | 27 | Report which of the following are publicly available and where they can be found: template data collection forms; data extracted from included studies; data used for all analyses; analytic code; any other materials used in the review. | 29 |

*From:*  Page MJ, McKenzie JE, Bossuyt PM, Boutron I, Hoffmann TC, Mulrow CD, et al. The PRISMA 2020 statement: an updated guideline for reporting systematic reviews. BMJ 2021;372:n71. doi: 10.1136/bmj.n71

**Supplemental S2-** Research Question, PICOTT and Search Strategy

**Research Question:** “Among adults with atrial fibrillation undergoing catheter ablation, do women versus men experience different postoperative outcomes (safety and efficacy) when compared within propensity score–matched observational studies?”

**PICOTT:**

**P – Population**

Patients diagnosed with **atrial fibrillation (AF)** (including paroxysmal, persistent, or long-standing persistent AF) who undergo **ablation** or pulmonary vein isolation.

**I – Intervention**

Ablation for atrial fibrillation performed in **women.**

**C – Comparison**

Ablation for atrial fibrillation performed in **men**.

**O – Outcomes**

*Primary outcomes include:*

- Freedom from AF/ atrial tachycardia (AT) recurrence
- Stroke/TIA
- All-cause mortality

*Secondary outcomes:*

- Procedural success rate / arrhythmia-free survival
- Recurrence of atrial fibrillation
- Procedural complications (e.g., cardiac tamponade, pericardial effusion, vascular or groin complications)
- Major bleeding
- Procedural mortality
- Risk of ACS
- Permanent pacemaker implantation (PPM)
- Pulmonary vein stenosis
- Fluoroscopy time
- Radiation dose
- Procedure duration or time
- Quality of life or symptom improvement
- Phrenic nerve injury

**T – Time**

Follow-up duration as reported in the included studies (e.g., **short-term ≤12 months** and **long-term >12 months** outcomes).

**T – Type of study**

**Randomized controlled trials (RCTs)**, **prospective cohort studies**, and **retrospective observational studies** comparing sex-based outcomes after ablation for atrial fibrillation.

**Search Strategy:**

**PubMed:** 896

("Atrial Fibrillation" OR "Atrial Fibrillation") AND ("Catheter Ablation" OR "Catheter Ablation" OR "Ablation" OR "Radiofrequency Ablation" OR "Cryoablation" OR "Pulmonary Vein Isolation" OR "PVI") AND ("Sex Factors" OR "Sex Characteristics" OR "Sex Distribution" OR "Sex Difference" OR "Gender Differences" OR "sex difference" OR "sex differences" OR "gender difference" OR "gender differences" OR "sex-based" OR "gender-based" OR "sex-specific" OR "gender-specific" OR "sex-related" OR "gender-related" OR "sex dimorphism" OR "sex dimorphic" OR "sex influence" OR "gender influence" OR "sex effect" OR "gender effect" OR "male vs female" OR "female vs male" OR "men vs women" OR "man vs woman" OR "male female" OR "female male" OR ("Sex" AND "Difference") OR ("Gender" AND "Difference") OR ("Sex" AND "Outcome") OR ("Gender" AND "Outcome"))

**Embase:**2273

("Atrial Fibrillation" OR "Atrial Fibrillation") AND ("Catheter Ablation" OR "Catheter Ablation" OR "Ablation" OR "Radiofrequency Ablation" OR "Cryoablation" OR "Pulmonary Vein Isolation" OR "PVI") AND ("Sex Factors" OR "Sex Characteristics" OR "Sex Distribution" OR "Sex Difference" OR "Gender Differences" OR "sex difference" OR "sex differences" OR "gender difference" OR "gender differences" OR "sex-based" OR "gender-based" OR "sex-specific" OR "gender-specific" OR "sex-related" OR "gender-related" OR "sex dimorphism" OR "sex dimorphic" OR "sex influence" OR "gender influence" OR "sex effect" OR "gender effect" OR "male vs female" OR "female vs male" OR "men vs women" OR "man vs woman" OR "male female" OR "female male" OR ("Sex" AND "Difference") OR ("Gender" AND "Difference") OR ("Sex" AND "Outcome") OR ("Gender" AND "Outcome"))

**Web of Science:** 724

("Atrial Fibrillation" OR "Atrial Fibrillation") AND ("Catheter Ablation" OR "Catheter Ablation" OR "Ablation" OR "Radiofrequency Ablation" OR "Cryoablation" OR "Pulmonary Vein Isolation" OR "PVI") AND ("Sex Factors" OR "Sex Characteristics" OR "Sex Distribution" OR "Sex Difference" OR "Gender Differences" OR "sex difference" OR "sex differences" OR "gender difference" OR "gender differences" OR "sex-based" OR "gender-based" OR "sex-specific" OR "gender-specific" OR "sex-related" OR "gender-related" OR "sex dimorphism" OR "sex dimorphic" OR "sex influence" OR "gender influence" OR "sex effect" OR "gender effect" OR "male vs female" OR "female vs male" OR "men vs women" OR "man vs woman" OR "male female" OR "female male" OR ("Sex" AND "Difference") OR ("Gender" AND "Difference") OR ("Sex" AND "Outcome") OR ("Gender" AND "Outcome"))

**Cochrane:** 239

("Atrial Fibrillation" OR "Atrial Fibrillation") AND ("Catheter Ablation" OR "Catheter Ablation" OR "Ablation" OR "Radiofrequency Ablation" OR "Cryoablation" OR "Pulmonary Vein Isolation" OR "PVI") AND ("Sex Factors" OR "Sex Characteristics" OR "Sex Distribution" OR "Sex Difference" OR "Gender Differences" OR "sex difference" OR "sex differences" OR "gender difference" OR "gender differences" OR "sex-based" OR "gender-based" OR "sex-specific" OR "gender-specific" OR "sex-related" OR "gender-related" OR "sex dimorphism" OR "sex dimorphic" OR "sex influence" OR "gender influence" OR "sex effect" OR "gender effect" OR "male vs female" OR "female vs male" OR "men vs women" OR "man vs woman" OR "male female" OR "female male" OR ("Sex" AND "Difference") OR ("Gender" AND "Difference") OR ("Sex" AND "Outcome") OR ("Gender" AND "Outcome"))

**Supplementary S3**- Forest plots for early secondary outcomes. (a) AV fistula (b) cardiac tamponade (c) complication rate (d) hematoma (e) pericardial effusion (f) phrenic nerve injury (g) pneumothorax or hemothorax (h) pneumonia (i) stroke/TIA (j) vascular complications (k) PVI success (l) AAD use at discharge

(a) Early AV fistula


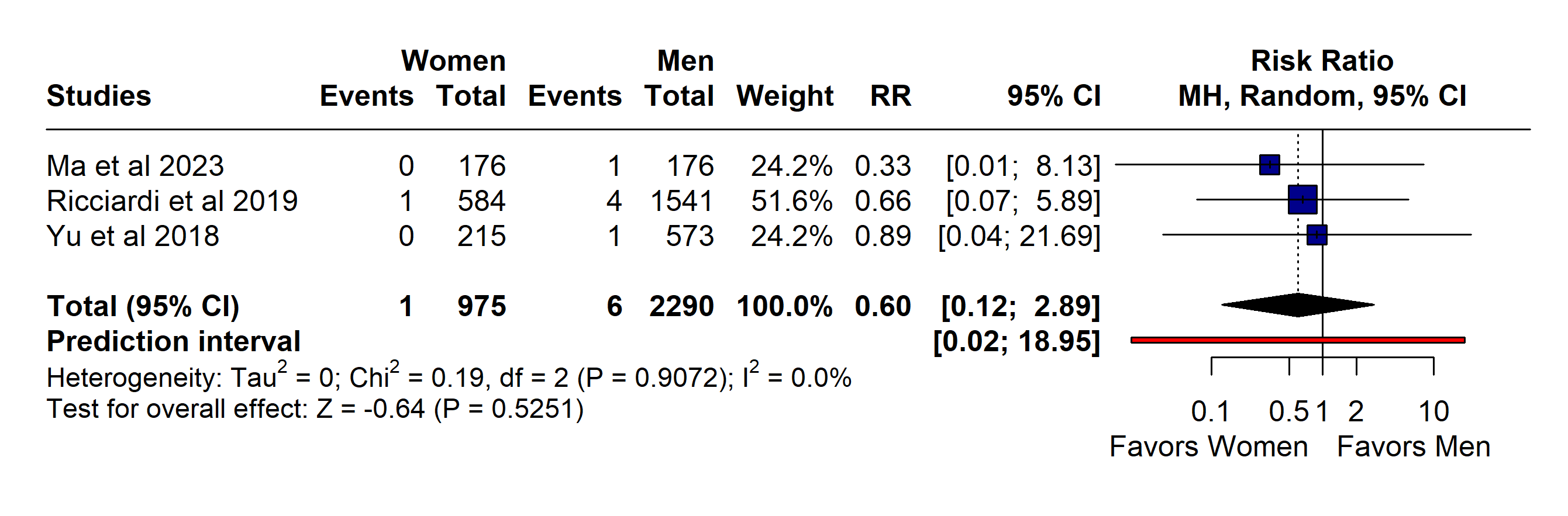


(b) Early cardiac tamponade


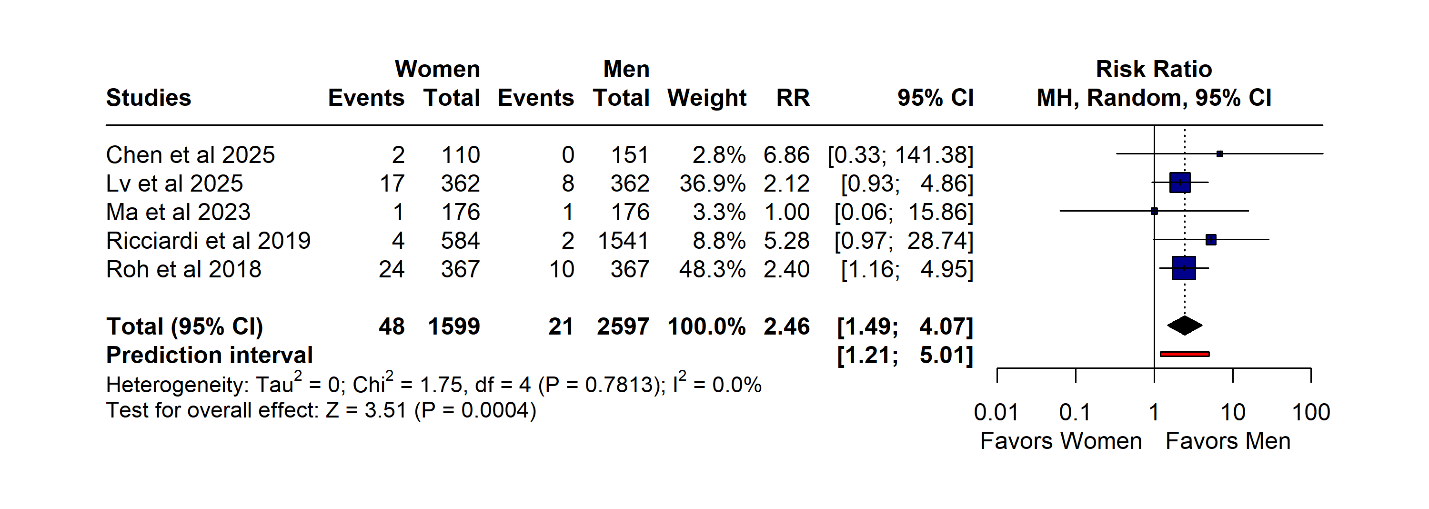


(c) Early complication rate


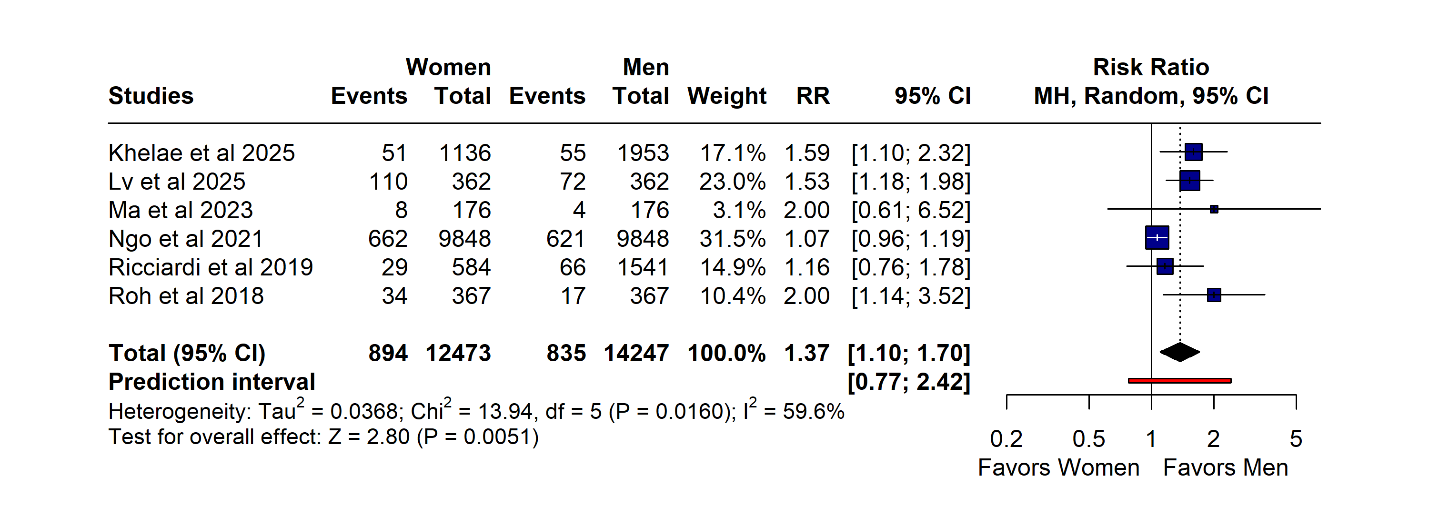


(d) Early hematoma


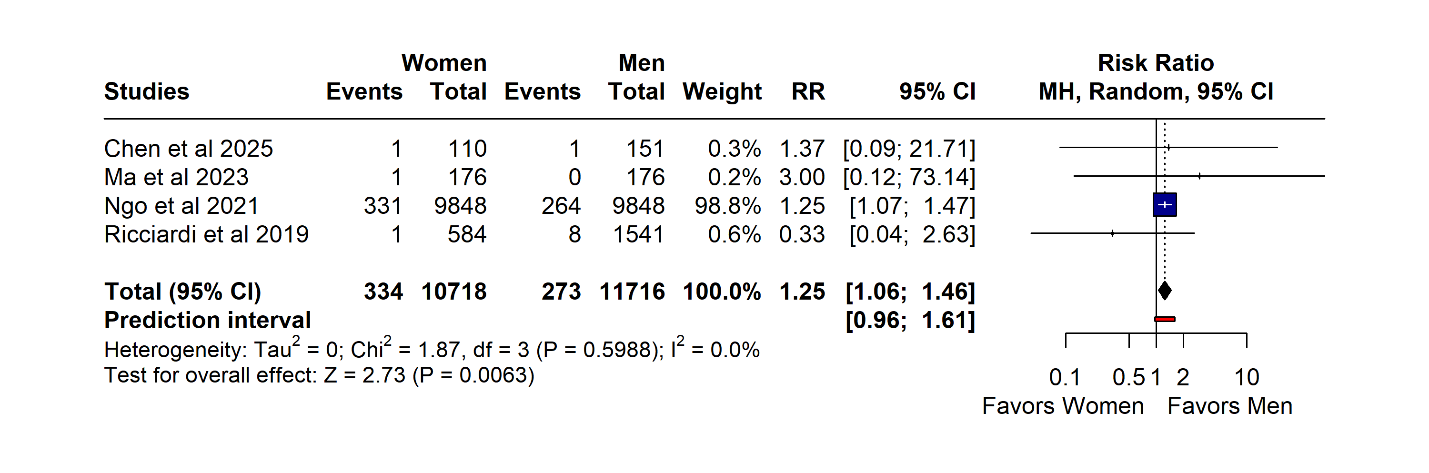


(e) Early pericardial effusion


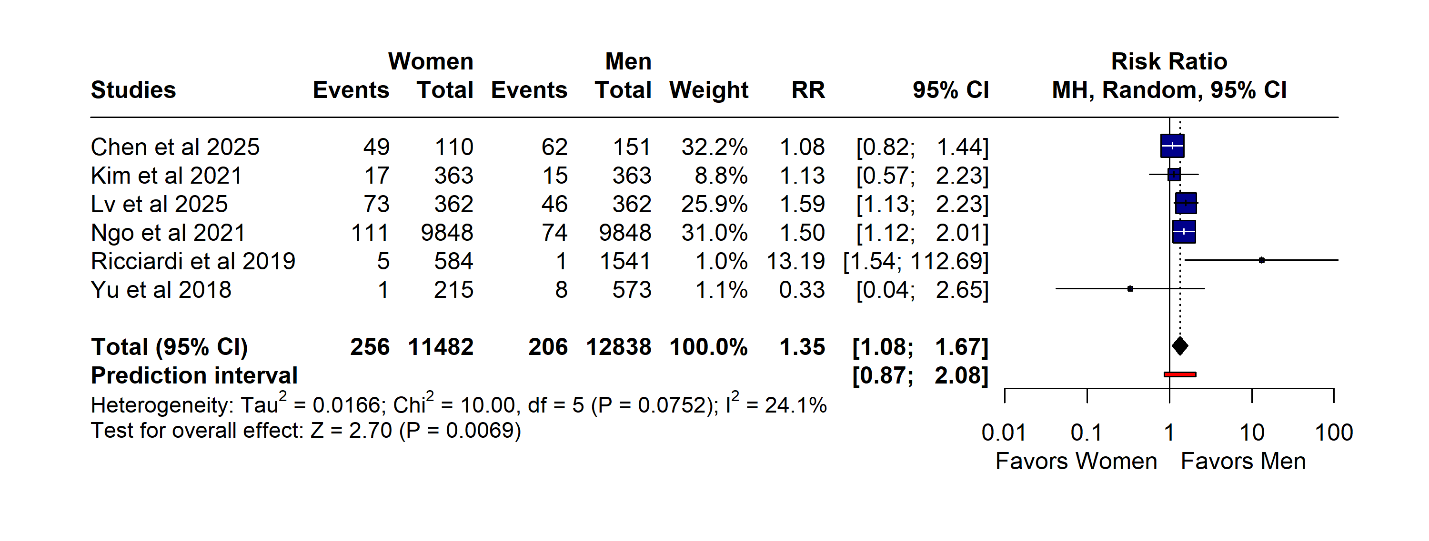


(f) Early phrenic nerve injury


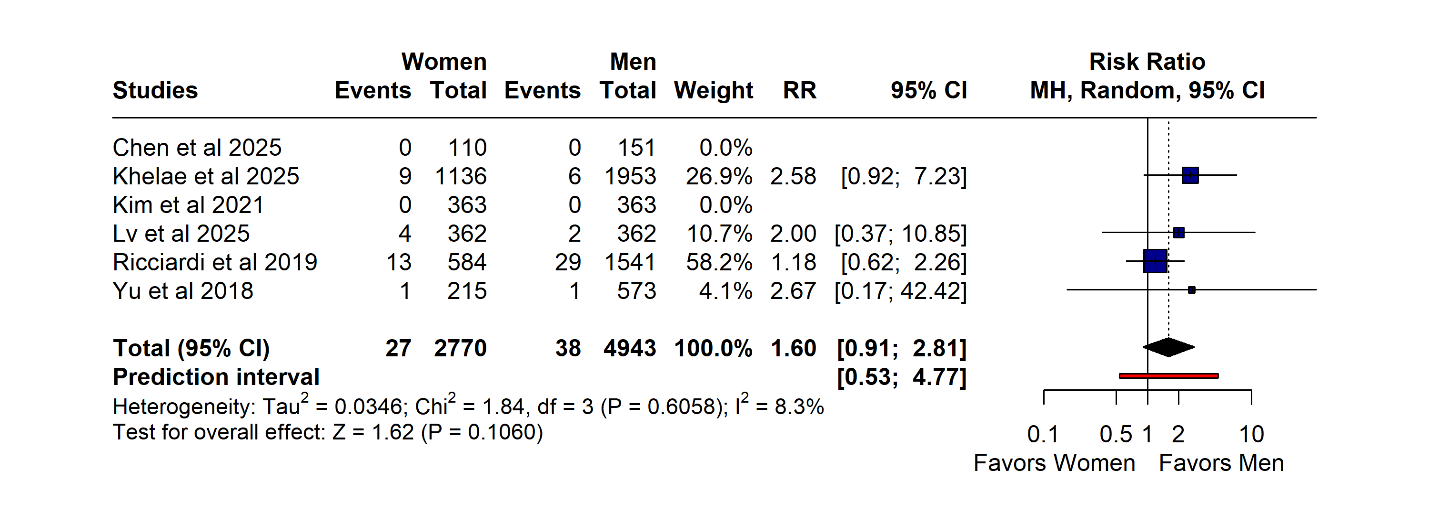


(g) Early pneumothorax or hemothorax


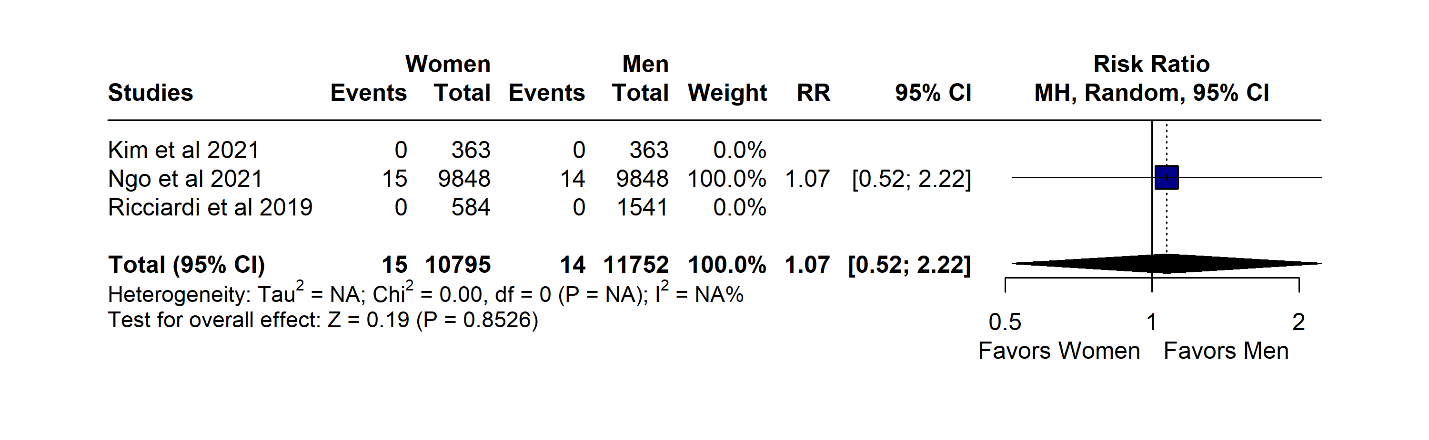


(h) Early pneumonia


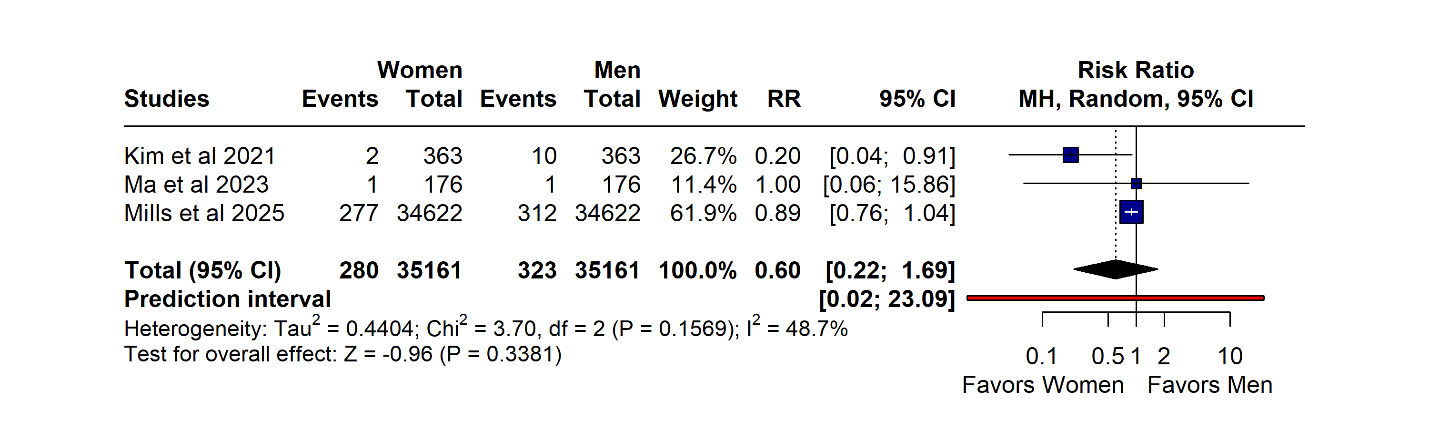


(i) Early stroke/TIA


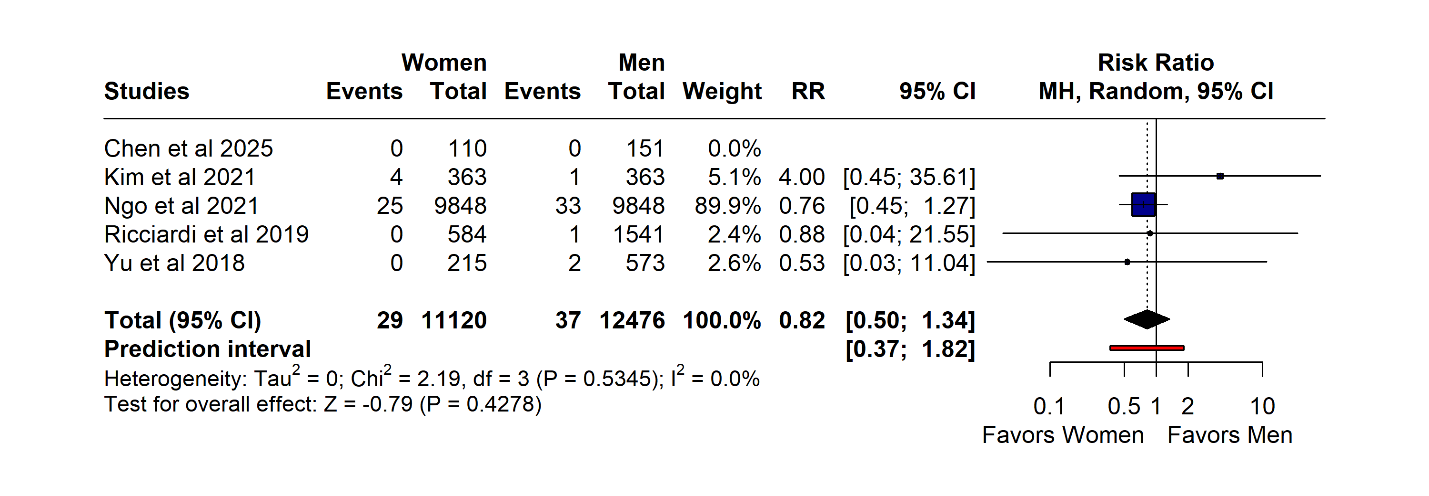


(j) Early vascular complications


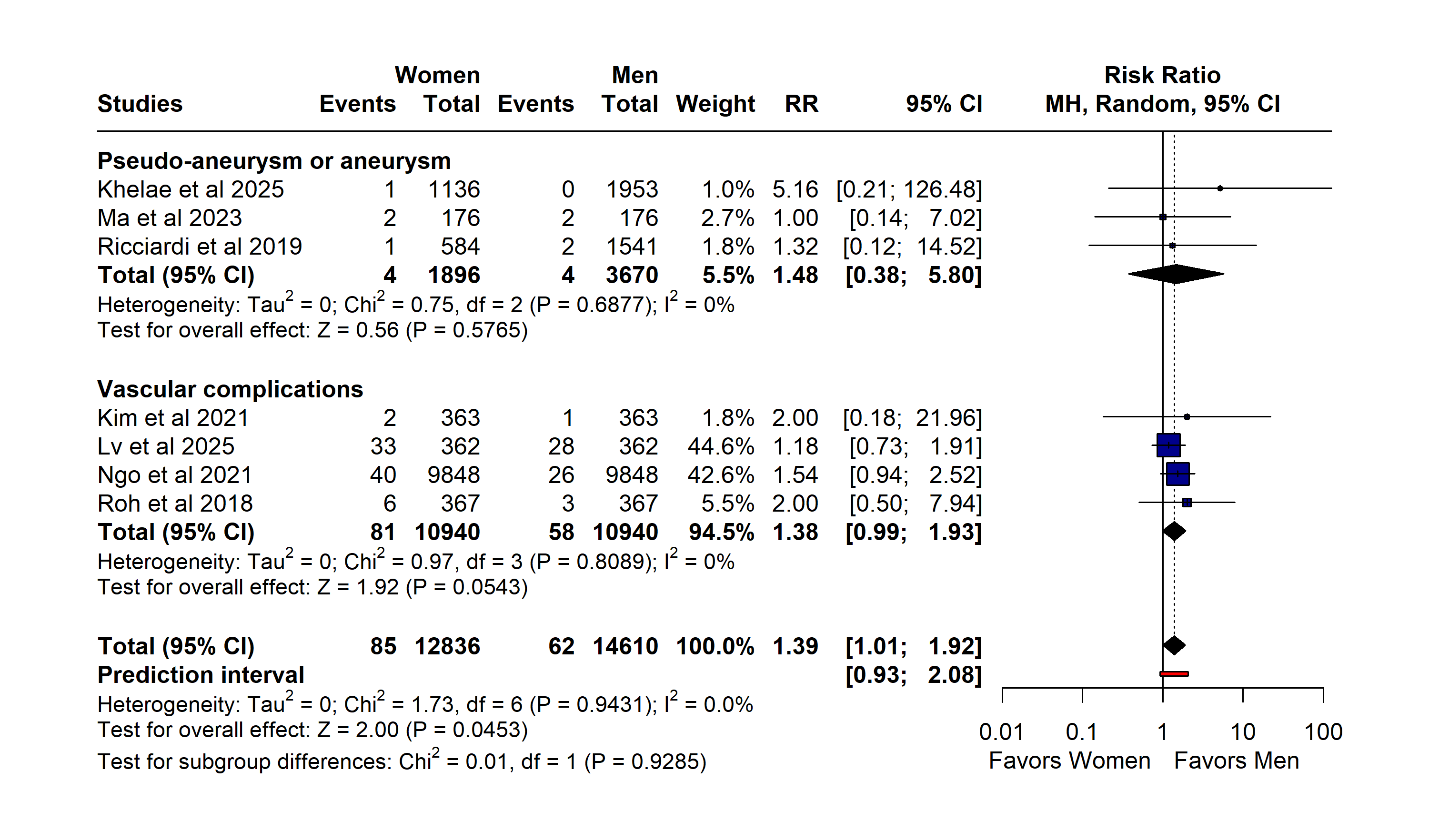


(k) Early PVI success


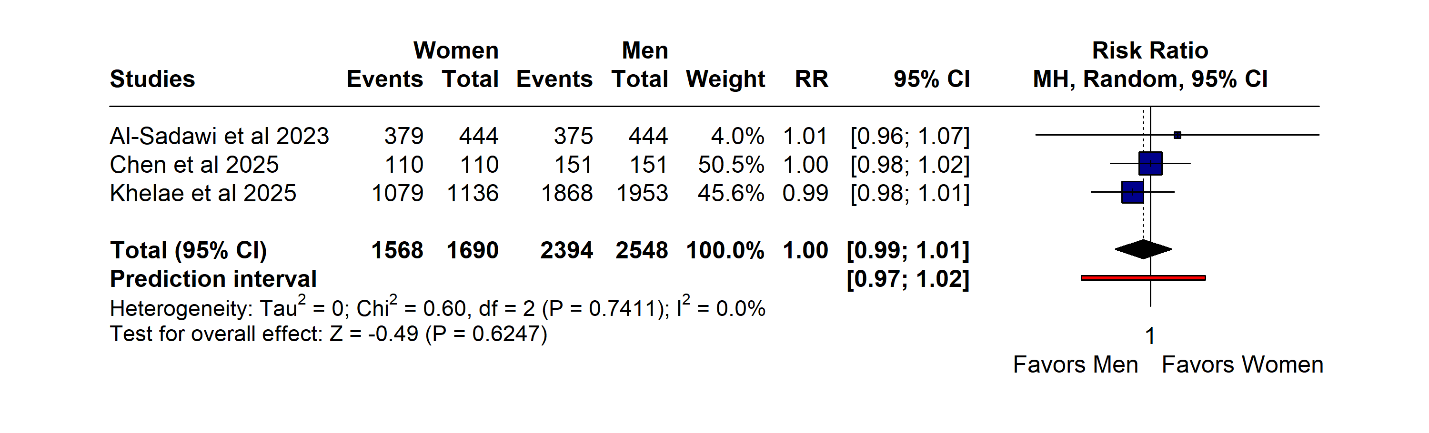


(l) AAD use at discharge


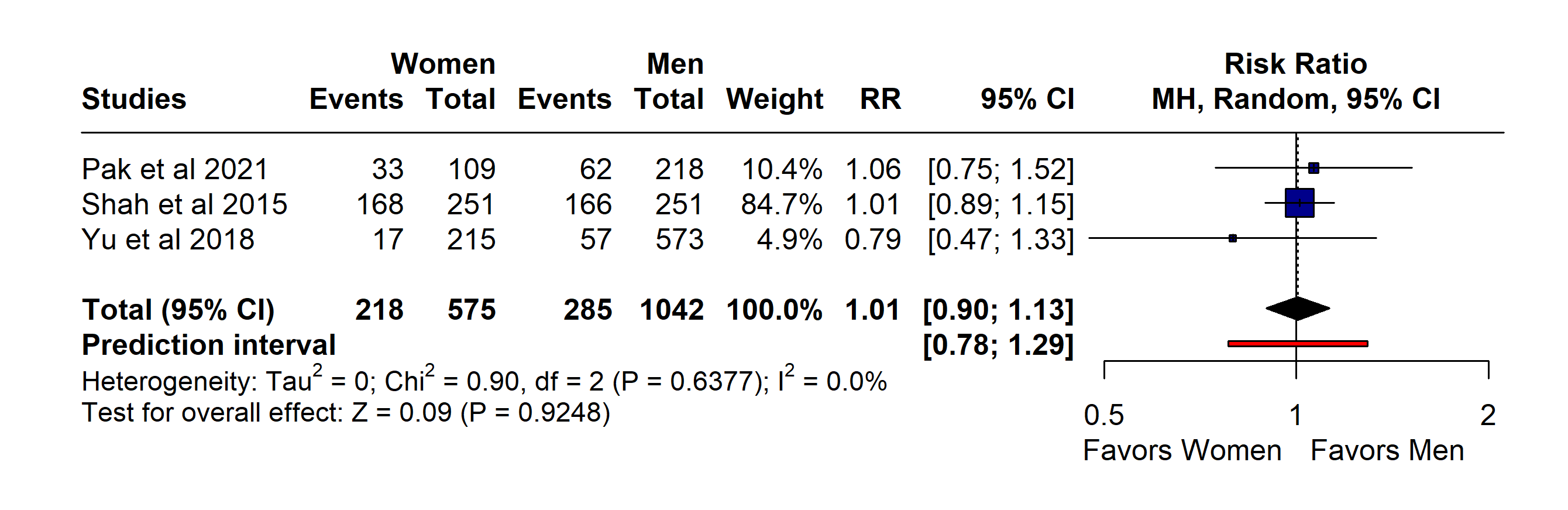


**Supplementary S4**- Forest plots for long-term secondary outcomes. (a) follow-up stroke/TIA (b) follow-up repeat ablation (c) follow-up cardiac failure (d) follow-up AAD

(a) follow-up stroke/TIA


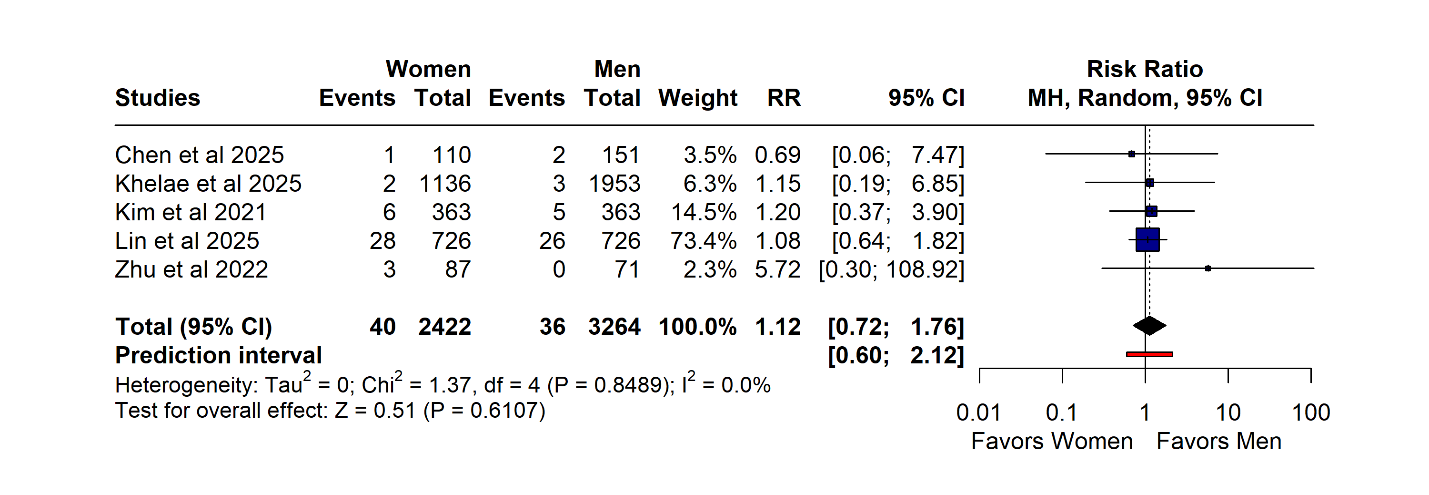


(b) follow-up repeat ablation


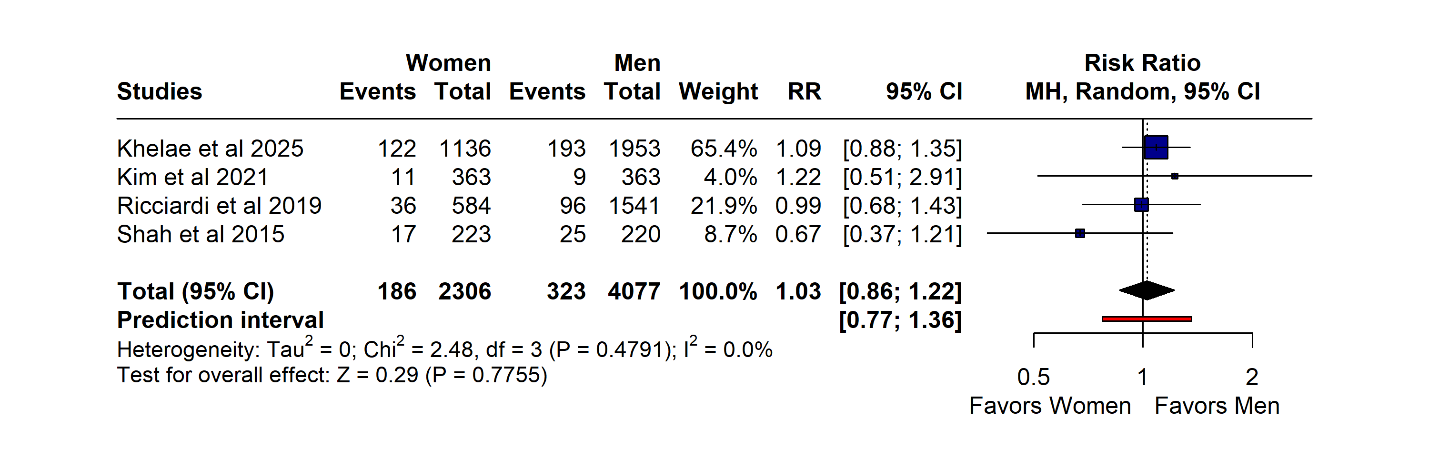


(c) follow-up cardiac failure


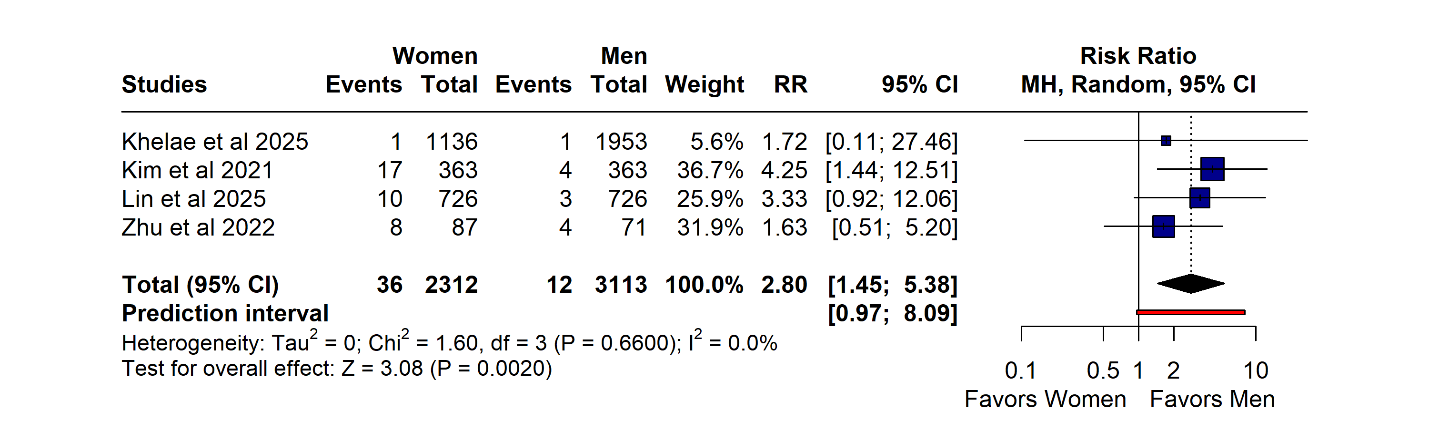


(d) follow-up AAD


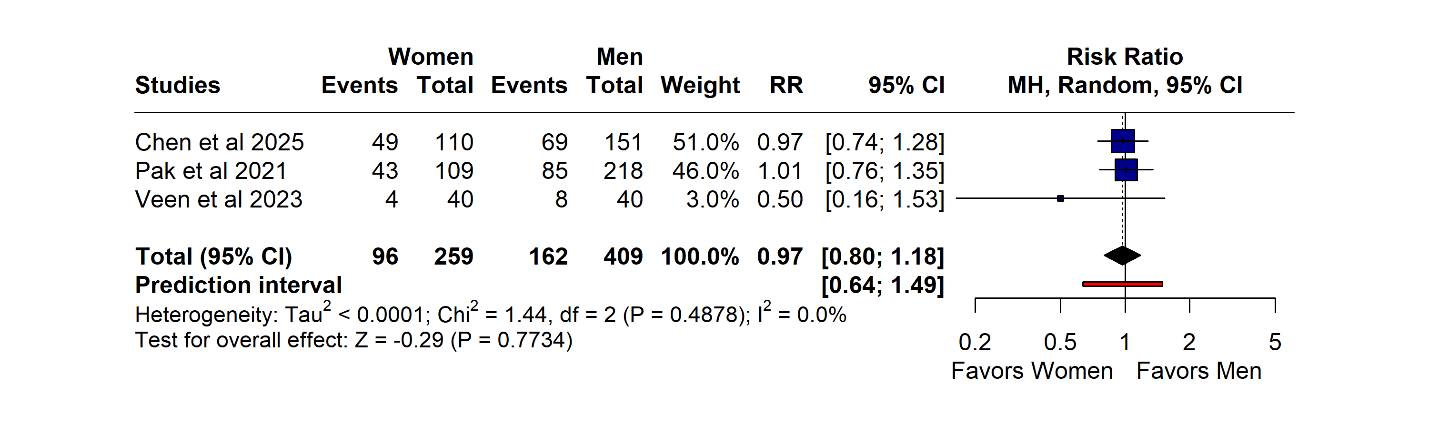


**Supplementary S5**- Leave one out sensitivity analysis. (a) atrial fibrillation (AF) recurrence at 1-year (b) atrial fibrillation / atrial flutter / atrial tachycardia (AF / AFL/ AT ) recurrence at 1-year (c) 1-year all-cause mortality (d) early complication rate (e) early pneumonia

(a) atrial fibrillation (AF) recurrence at 1-year


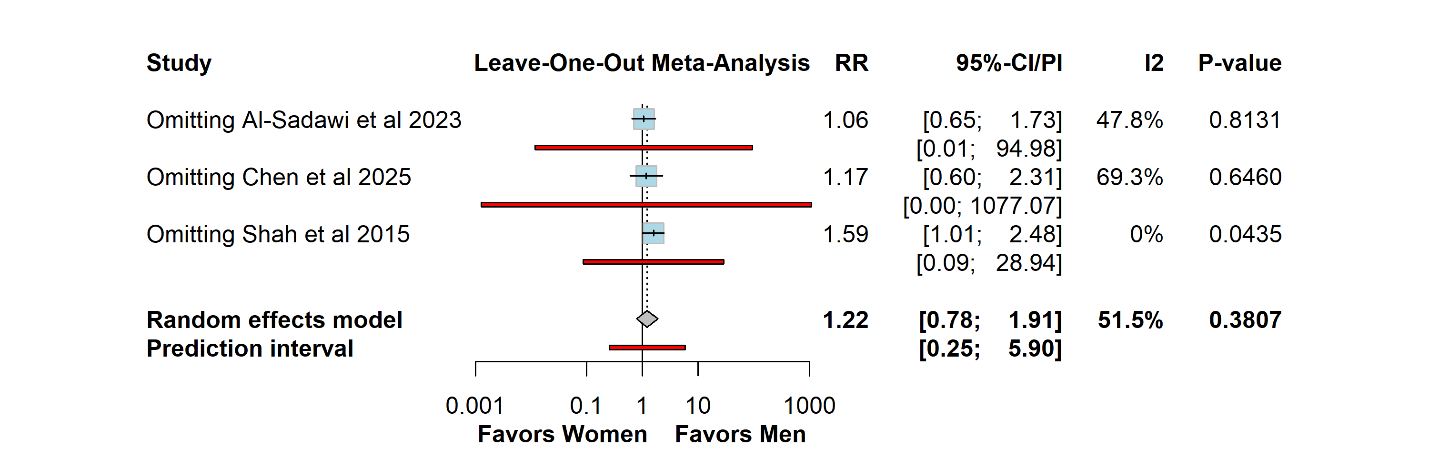


(b) atrial fibrillation / atrial flutter / atrial tachycardia (AF / AFL/ AT ) recurrence at 1-year


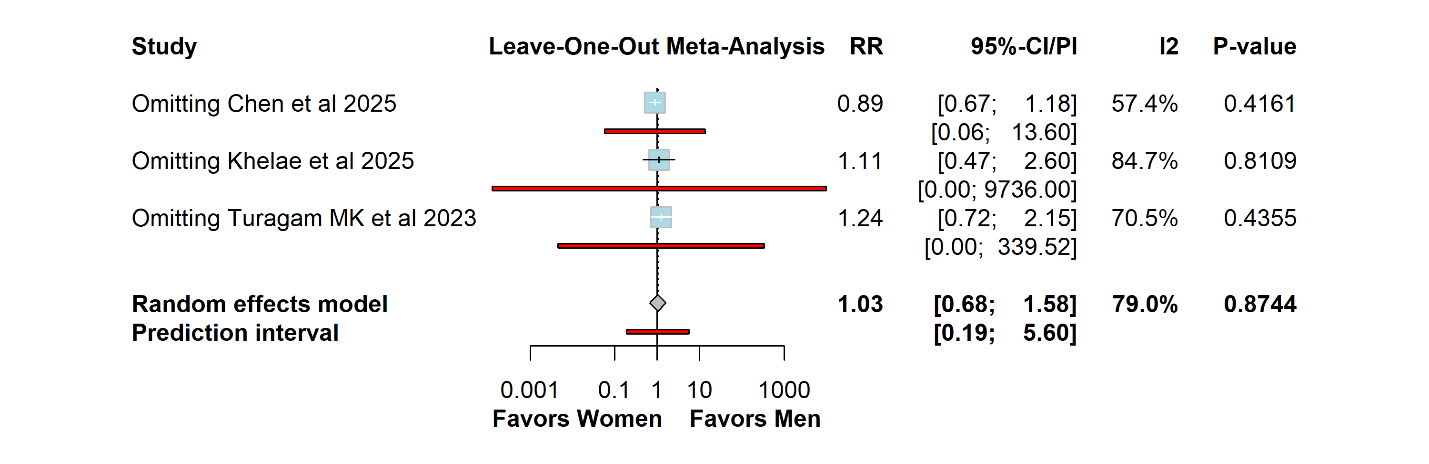


(c) 1-year all-cause mortality


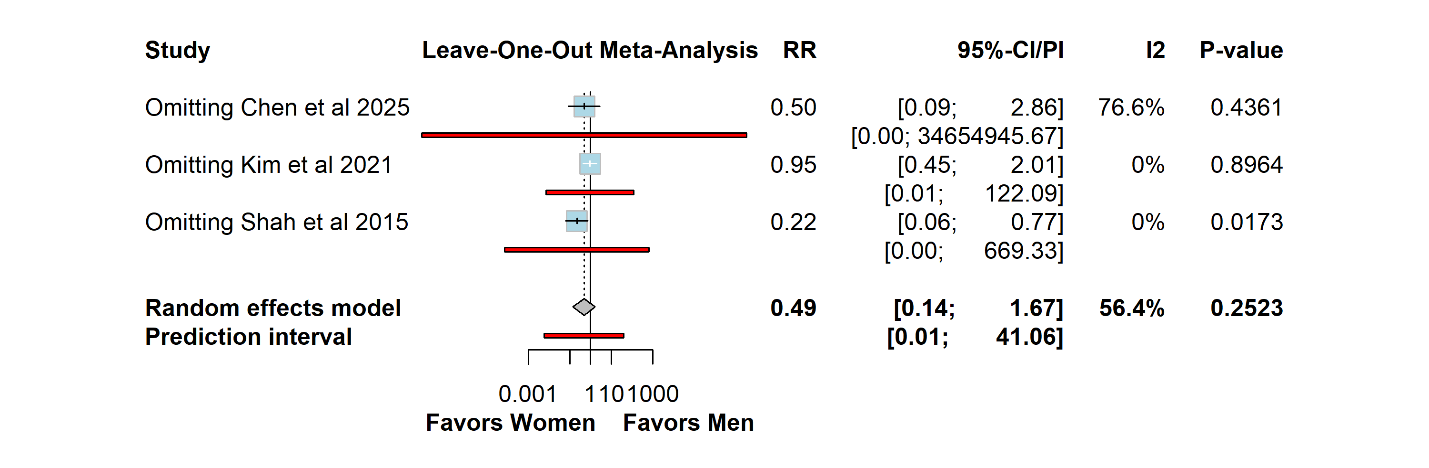


(d) early complication rate


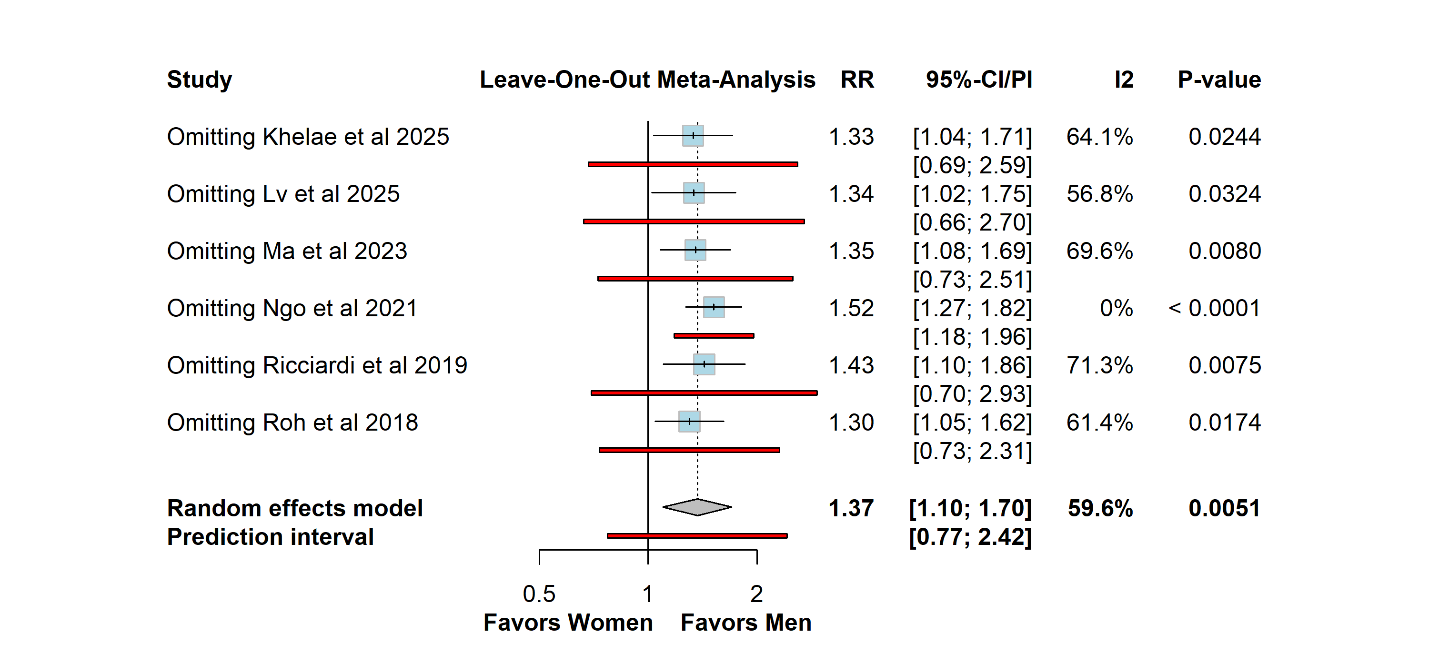


(e) early pneumonia


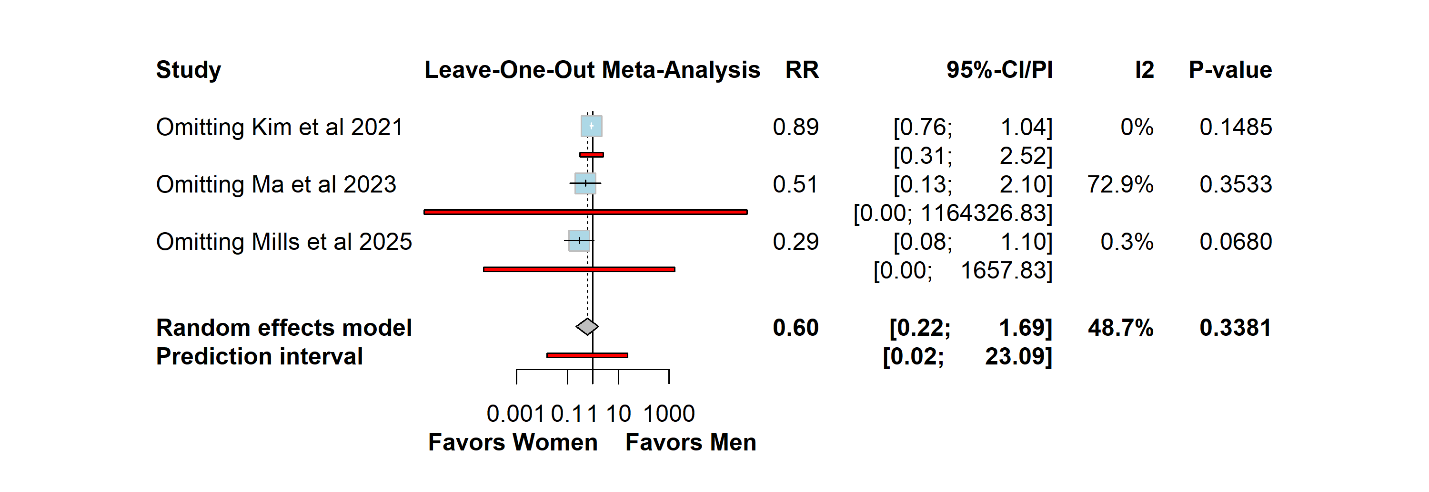


**Supplementary S6-** GRADE certainty assessment

**CI:** confidence interval; **RR:** risk ratio

| **Female compared to Male in catheter ablation for atrial fibrillation**  **Bibliography:** | | | | | | | | | | | |
| --- | --- | --- | --- | --- | --- | --- | --- | --- | --- | --- | --- |
| **Certainty assessment** | | | | | | | **Summary of findings** | | | | |
| **Participants  (studies)  Follow-up** | **Risk of bias** | **Inconsistency** | **Indirectness** | **Imprecision** | **Publication bias** | **Overall certainty of evidence** | **Study event rates (%)** | | **Relative effect  (95% CI)** | **Anticipated absolute effects** | |
|  |  |  |  |  |  |  | **With Male** | **With Female** |  | **Risk with Male** | **Risk difference with Female** |
| **AF recurrence (one-year)** | | | | | | | | | | | |
| 714  (3 non-randomised studies) | very serious^a^ | very serious^b^ | not serious | not serious | none | ⨁◯◯◯  Very low^a,b^ | 69/379 (18.2%) | 73/335 (21.8%) | **RR 1.22**  (0.78 to 1.91) | 69/379 (18.2%) | **40 more per 1000**  (from 40 fewer to 166 more) |
| **AF or AT recurrence (one-year)** | | | | | | | | | | | |
| 3326  (4 non-randomised studies) | very serious^a^ | not serious | not serious | not serious | none | ⨁⨁◯◯  Low^a^ | 183/2139 (8.6%) | 146/1187 (12.3%) | **RR 1.13**  (0.91 to 1.39) | 183/2139 (8.6%) | **11 more per 1000**  (from 8 fewer to 33 more) |
| **AF or AT recurrence (two-year)** | | | | | | | | | | | |
| 3289  (3 non-randomised studies) | very serious^a^ | not serious | not serious | not serious | none | ⨁⨁◯◯  Low^a^ | 313/2123 (14.7%) | 245/1166 (21.0%) | **RR 1.22**  (1.04 to 1.42) | 313/2123 (14.7%) | **32 more per 1000**  (from 6 more to 62 more) |
| **AF or AT or AFL recurrence (one-year)** | | | | | | | | | | | |
| 4080  (3 non-randomised studies) | very serious^a^ | very serious^b^ | not serious | not serious | none | ⨁◯◯◯  Very low^a,b^ | 340/2469 (13.8%) | 221/1611 (13.7%) | **RR 1.03**  (0.68 to 1.58) | 340/2469 (13.8%) | **4 more per 1000**  (from 44 fewer to 80 more) |
| **All-cause mortality (30-day)** | | | | | | | | | | | |
| 21407  (4 non-randomised studies) | very serious^a^ | not serious | not serious | not serious | none | ⨁⨁◯◯  Low^a^ | 13/10724 (0.1%) | 14/10683 (0.1%) | **RR 1.07**  (0.50 to 2.29) | 13/10724 (0.1%) | **0 fewer per 1000**  (from 1 fewer to 2 more) |

#### **Explanations**

a. Serious risk of bias

b. Serious inconsistency

**Supplementary S7-** Quality assessment using ROBINS-I


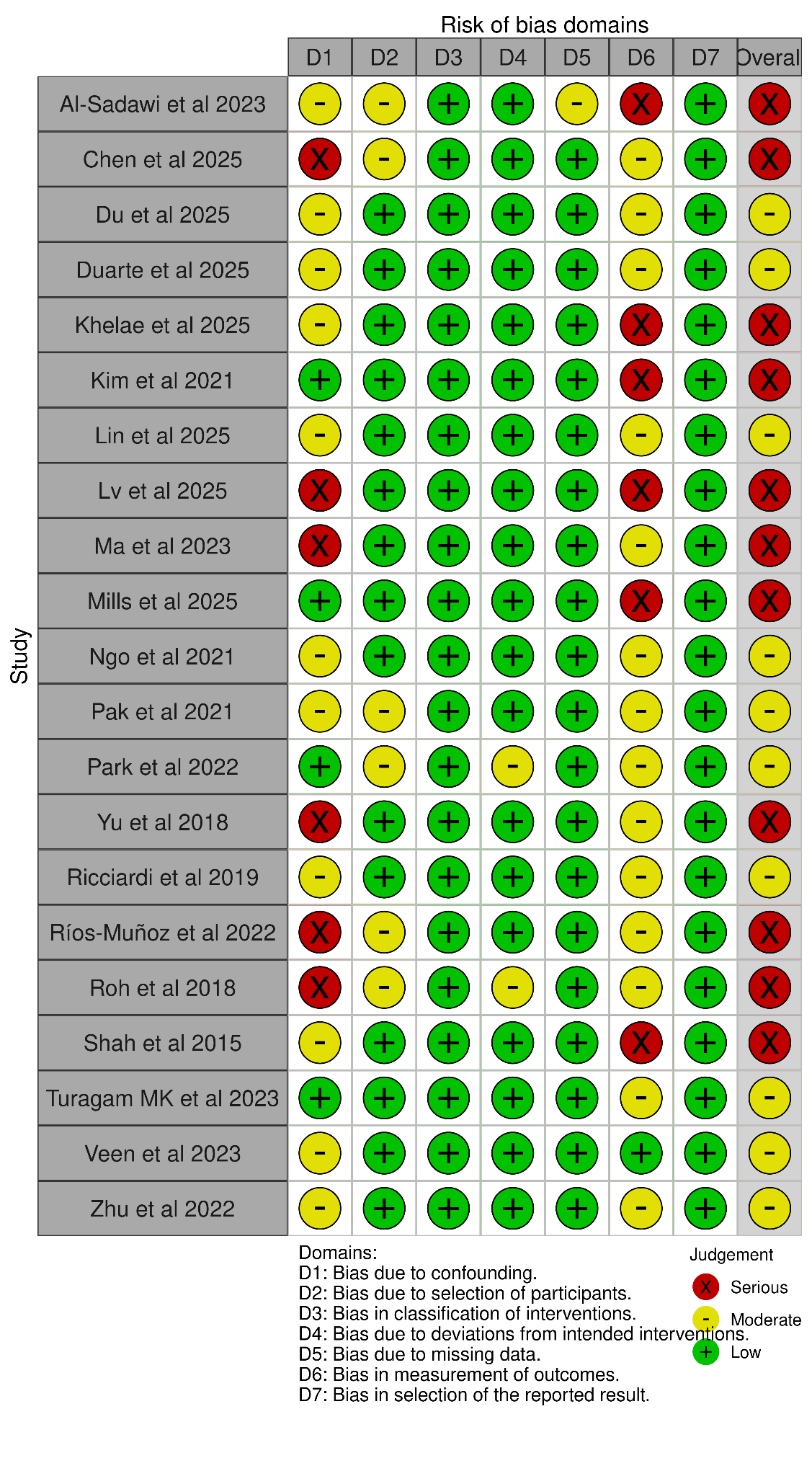


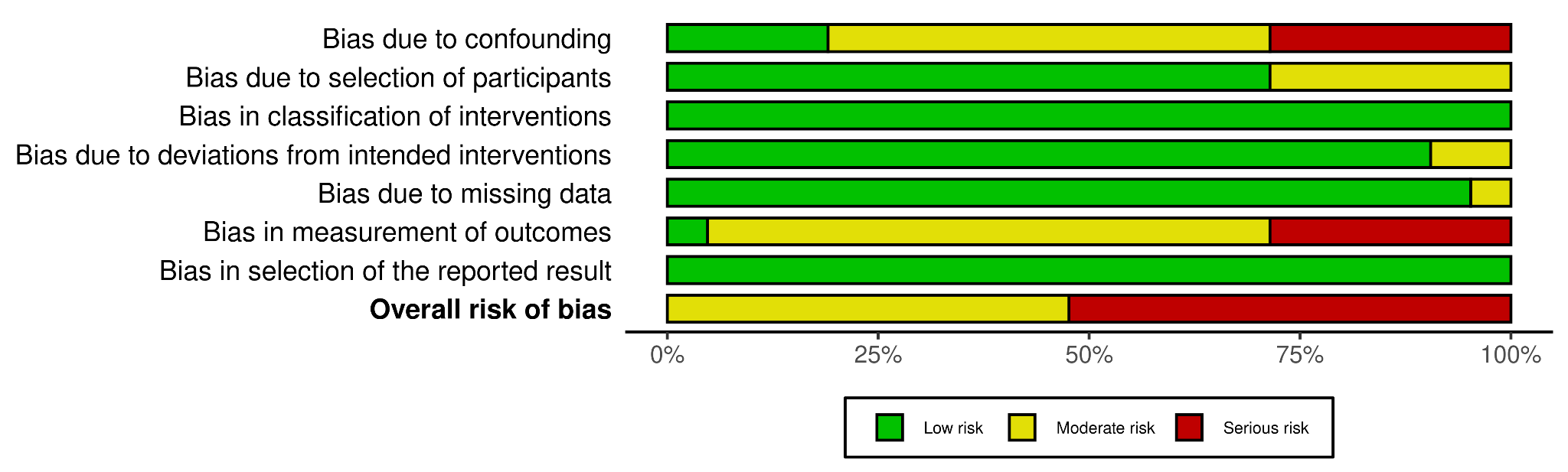


**Supplementary S8-** Propensity Score Matching (PSM) Protocol Summary Table

**Supplementary Table : PSM summary table**

| Study ID | PSM | variables | ratio | Female / Male |
| --- | --- | --- | --- | --- |
| Al-Sadawi et al 2023 | PSM–analysis was performed based on previously published papers in the literature about the main factors affecting AF ablation outcomes. | sex, age, BMI, race, HTN, DM | 1 : 1 | 444 / 444 |
| Chen et al 2025 | PSM was used to reduce treatment‐selection bias and potential confounding. We calculated a propensity score  of 0.1 for maximum execution performance and fixed caliper width. | age, heart failure,HTN, HAS-BLED score, NT-proBNP , GFR below 60, anticoagulation, echocardiographic measurements, EIVOM success and ablation strategy which were possibly related to AF recurrence | 1:1.5 | 110 / 151 |
| Du et al 2025 | To account for potential confounding variables, PSM with a nearest neighbor matching algorithm was performed as a sensitivity analysis. A 1:1 female-to-male matching was implemented using a caliper value of 0.02 to balance covariate distributions. | age, AF duration, comorbidities, extra-PV ablation and LAVI | 1 : 1 | 271 / 271 |
| Duarte et al 2025 | PSM analysis was performed using a multivariate logistic regression model where sex was modeled with the same 11 baseline variables included in the regression analysis. Matching employed a 1:1 nearest neighbor method with a greedy matching algorithm and a caliper width of 0.2 standard deviations of the PS logit (0.18), using the R package Matchlt. Post-PS matching, absolute standardized mean differences between groups were assessed for all baseline variables. | age, BMI, years since AF diagnosis, HTN, heart failure, type of AF, CKD, CAD, CVD, DM, Ablation energy | 1 : 1 | 113 / 113 |
| Khelae et al 2025 | Logistic regression was used to calculate propensity score for each subject where group cohort (female vs. male) was the dependent variable, and baseline variables from Table 1 Multiple imputation methods were utilized in the propensity score modeling, imputing baseline data for patients missing data. Multivariate imputation by fully conditional specification methods were utilized with logistic regression method specified for classification variables and regression methods utilized for continuous variables | age, BMI, type of AF (paroxysmal vs.  persistent), years diagnosed with AF, history of atrial flutter,  history of atrial tachycardia, LAD, LVEF, number of prior failed AADs, HTN, baseline NYHA, prior MI, prior stroke/TIA, CAD, DM and sleep apnea were included  as covariates. CHA2DS2-VASc score was excluded as it is a  composite of other variables in the model (heart failure,  hypertension, age, diabetes, prior stroke/TIA). | NA | 1136 / 1953 |
| Kim et al 2021 | For sensitivity analysis, 1:1 propensity score matching was used. A propensity score was estimated using logistic regression based on socio-demographics, medical history, concurrent medication use, and AF duration | socio-demographics, medical history, concurrent medication use, and AF duration | 1 : 1 | 363 / 363 |
| Lin et al 2025 | To balance the baseline clinical characteristics between sexes, we performed a 1:1 PSM. | age ≥60 years, obesity, AF duration since diagnosis, HTN, stroke, vascular disease, CHA2DS2-VASc score above (including risk factors other than female), AF type, and LAD ≥40 mm. | 1 : 1 | 726 / 726 |
| Lv et al 2025 | Used R4.0.0 to carry out 1:1 PSM analysis, calculated the tendency score, with a caliper value of 0.1, and matched according to the nearest neighbor matching method. | age, ethnicity, AF type, BMI, NYHA, CHA2DS2-VASc points, HF, CAD, HTN, DM, embolism history, HCM, OSAHS, history of cardiac surgery, albumin, AST, ALT, creatinine, LVEF, LAD, RAD | 1 : 1 | 362 / 362 |
| Ma et al 2023 | PSM was used to adjust for demographic characteristics. This method allowed pseudo-randomness to select patients with similar characteristics by using the 1:1 nearest neighbor matching method. The score was based on a logistic regression model. The reasons we controlled these characteristics were: (1) women are usually older than men when they undergo catheter ablation, which may bring differences in concomitant diseases or AF recurrence risk; (2) women often receive catheter ablation later than men, which may bring differences in ablation sites or AF recurrence risk; (3) BMI is associated with more procedure-related complications. | age, BMI, and AF duration. | 1 : 1 | 176 / 176 |
| Mills et al 2025 | Propensity‐score matching (PSM) was performed to balance baseline differences between the two groups. TriNetX uses a built‐in logistic regression algorithm to generate propensity‐scores, followed by greedy nearest neighbor matching with a caliper of 0.1 pooled standard deviations to identify the matched subsets. Female and male patients were 1:1 propensity‐score matched for age, comorbidities, and medications. | age, co‐morbidities (AF type, IHD, HTN, DM, COPD, asthma, IBD, CKD, obesity, thyroid dysfunctions ), medications (  beta‐blocker, flecainide , amiodarone, anti‐anginal medication, antacids, omeprazole, laxatives) | 1 : 1 | 34622 / 34622 |
| Ngo et al 2021 | A propensity score, indicating the conditional probability that any patient would be female, was estimated using a non-parsimonious logistic regression model.Each female patient was then matched 1:1 without replacement to the closest male patient based on the propensity score using a calliper width of 0.01 to form the matched cohort. Matching quality was evaluated by estimating the standardized bias with values of <_5% indicating good covariate balance. Logistic regression was applied on these matched pairs with female gender as the only independent variable to evaluate its association with the risk of complications. | age, history of AF hospitalizations and AF ablation in the preceding year, ablation in both atria, year of ablation, total length of stay of the index hospitalization, and 180 comorbidities derived from the Condition Categories system. | 1 : 1 | 9848 / 9848 |
| Pak et al 2021 | We performed propensity score matching without a replacement and with a calliper of 0.1 at a female to male ratio of 1:2 . | age and AF type | 1:2 | 109 / 218 |
| Park et al 2022 | To adjust the selection bias between men and women, we conducted PSM analyses. For de novo AFCA patients, we performed PS matching with a calliper 0.25 and without replacement in 1:1 ratio. | age, AF type, BSA, LAD, LVEF, the ratio of the early diastolic mitral inflow velocity to the early mitral annular velocity (E/Em) and heart failure. | 1 : 1 | 469 / 469 |
| Ricciardi et al 2019 | To account for differences in baseline characteristics between men and women, PS methods were utilized to estimate an adjusted HR for efficacy between men and women. | The PS method was used to adjust the group’s HR in multivariable Cox analysis; the Cox multivariable model included propensity score (as a continuous variable), age, and AF type as covariates in the model. | NA | 584 / 1541 |
| Ríos-Muñoz et al 2022 | A propensity score matching was applied to reduce the effect of baseline differences in the data population by gender. We ran a logistic regression on the gender group and as covariates for the analysis, we selected those that were significantly different (p<0.05) in Table 1. | age, HTN, BSA, and CHA2DS2-VASc. | NA | 21 / 16 |
| Roh et al 2018 | We considered index procedure era as matching criteria to decrease bias related with ablation technology development. | age (±1 year), type of AF (paroxysmal or non-paroxysmal), and duration of AF before index procedure (±2 years) | 1 : 1 | 367 / 367 |
| Shah et al 2015 | To account for baseline differences between women and men, 1:1 PSM methods were used, with a caliper of size 0.02 logit propensity score SD units. For each baseline covariate included in the propensity-score model, balance between genders was assessed using standardized differences. | age, BMI, aortic, mitral, or tricuspid valve surgery, CAD, family history of CAD, DM, hypercholesterolemia, HTN, prior MI, prior CABG, prior valve surgery; prior cardiovascular intervention; prior congestive heart failure, NYHA class III/IV, repeat sternotomy, elective status, and preoperative medication (lipid-lowering drugs,AADs) | 1 : 1 | 251 / 251 |
| Turagam MK et al 2023 | PSM was performed | age, BMI, CAD, heart failure, HTN, sleep apnea, and DM | 1 : 1 | 365 / 365 |
| Veen et al 2023 | A PSM analysis was performed, using logistic regression which was based on age and BMI. The (nearest) neighboring propensity score with a match tolerance of 0.05 determined the random assignment of cases to controls. | age, BMI | 1 : 1 | 40 / 40 |
| Yu et al 2018 | Propensity scores were used to match men to women to reduce the potential confounding in this observational study. Propensity scores were estimated using a non-parsimonious multiple logistic regression model for men and women. A matching caliper of 0.1 standard deviations of the logit of the estimated propensity score was enforced to ensure that matches of poor fit were excluded. Cases were then matched, without replacement, with controls based on the closest possible value of the propensity score (nearest neighbor matching). | age, type of AF, and the comorbidities that make up the CHA2DS2-VASc score, which is also known as an important score associated with recurrence after AF ablation. | NA | 215 / 573 |
| Zhu et al 2022 | The postmenopausal women and men matched for age, BMI, and type of AF were included. | age, BMI, and type of AF | NA | 87 / 71 |

AAD – antiarrhythmic drugs, AF – atrial fibrillation, BMI – body mass index, CAD – coronary artery disease, CKD – chronic kidney disease, CVD – cerebrovascular disease, DM – diabetes mellitus, EIVOM – ethanol infusion into the vein of Marshall, GFR – glomerular filtration rate, HAS-BLED score – hypertension, abnormal renal/liver function, stroke, bleeding history or predisposition, labile INR, elderly, drugs/alcohol score, HF – heart failure, HCM – hypertrophic cardiomyopathy, HTN – hypertension, LAD – left atrial diameter, LAVI – left atrial volume index, LVEF – left ventricular ejection fraction, NT-proBNP – N-terminal B-type natriuretic peptide, OSAHS – obstructive sleep apnea–hypopnea syndrome, PV – pulmonary vein.
